# Influenza in the school-aged population in Mexico: burden of disease and cost-effectiveness of vaccination in children

**DOI:** 10.1186/s12879-020-4948-5

**Published:** 2020-03-20

**Authors:** Jorge Abelardo Falcón-Lezama, Rodrigo Saucedo-Martínez, Miguel Betancourt-Cravioto, Myrna María Alfaro-Cortes, Roberto Isaac Bahena-González, Roberto Tapia-Conyer

**Affiliations:** 1 Sociedad Mexicana de Salud Pública, Herschel 109, Anzures, Miguel Hidalgo, 11590 Mexico City, Mexico; 2 Universidad Nacional Autónoma de México, Av. Universidad 3000, Circuito Escolar CU, Edificio B 1er Piso, Coyoacan, 04510 Mexico City, Mexico

**Keywords:** Influenza, Vaccination, School-aged population, Cost effectiveness, Mexico, Burden of disease, Middle-income countries

## Abstract

**Background:**

The current national influenza vaccination schedule in Mexico does not recommend vaccination in the school-aged population (5–11 years). Currently, there are limited data from middle-income countries analysing the cost-effectiveness of influenza vaccination in this population. We explored the clinical effects and economic benefits of expanding the current national influenza vaccination schedule in Mexico to include the school-aged population.

**Methods:**

A static 1-year model incorporating herd effect was used to assess the cost-effectiveness of expanding the current national influenza vaccination schedule of Mexico to include the school-aged population. We performed a cross-sectional epidemiological study using influenza records (2009–2018), death records (2010–2015), and discharge and hospitalisation records (2010–2016), from the databases of Mexico’s Influenza Surveillance System (SISVEFLU), the National Mortality Epidemiological and Statistical System (SEED), and the Automated Hospital Discharge System (SAEH), respectively. Cost estimates for influenza cases were based on 7 scenarios using data analysed from SISVEFLU; assumptions for clinical management of cases were defined according to Mexico’s national clinical guidelines. The primary health outcome for this study was the number of influenza cases avoided. A sensitivity analysis was performed using conservative and optimistic parameters (vaccination coverage: 30% / 70%, Vaccine effectiveness: 19% / 68%).

**Results:**

It was estimated that expanding the influenza immunisation programme to cover school-aged population in Mexico over the 2018–2019 influenza season would result in 671,461 cases of influenza avoided (50% coverage and 50% effectiveness assumed). Associated with this were 262,800 fewer outpatient consultations; 154,100 fewer emergency room consultations; 97,600 fewer hospitalisations, and 15 fewer deaths. Analysis of cases avoided by age-group showed that 55.4% of them were in the school-aged population, and the decrease in outpatient consultations was largest in this population. There was an overall decrease in the economic burden for the Mexican health care system of 111.9 million US dollars; the immunization programme was determined to be cost-saving in the base, conservative and optimistic scenarios.

**Conclusions:**

Vaccinating school-aged population in Mexico would be cost-effective; expansion of the current national vaccination schedule to this age group is supported.

## Background

Since isolation of the first influenza virus in the mid 1930’s [1], control of the virus has been a long-pursued global public health goal. From the early inactivated vaccines to the current recombinant quadrivalent vaccines, there is no doubt that science and technology have stepped up to this challenge [2–4]. However, the constant genetic shifts and drifts of the virus remain the most relevant factors hindering efforts at disease control; the current annual incidence of influenza-like illness (ILI) in Latin America ranges between 4.7 and 15.4% [5].

Vaccination is one of the most cost-effective strategies for disease prevention and control [6]. For influenza, the implementation of immunisation campaigns throughout the world have resulted in decreases of both mortality and morbidity [7, 8]. Although usually focused on younger populations for epidemiological and practical reasons, vaccination in most age groups, including adults, is considered highly cost-effective [9, 10].

Throughout the Americas, immunisation recommendations for children vary. According to the World Health Organization (WHO) Vaccine-Preventable Diseases Monitoring System, only Grenada, Panama, and the United States recommend universal influenza vaccination in children and adolescents [11]; Canada recommends immunisation for all children aged 6 to 59 months, and for at-risk children and adolescents [12].

In Mexico, the current national vaccination schedule recommends yearly immunisation in several target groups: children aged 6 to 59 months, adults aged > 60 years, pregnant women, at-risk individuals aged 5 to 59 years, and health professionals. These target groups are eligible to receive the vaccine free of charge at any public health facility during the influenza immunisation season (October to February). Vaccination of adults aged 50 to 59 years and school-aged children (5 to 11 years) who are not at risk are not considered as target groups for influenza immunisation [13].

Studies have shown that the cost-effectiveness of influenza vaccination varies depending on the age-groups targeted [14]. De Waure et al. assessed the economic benefits of influenza vaccination and found that most studies they reviewed analysed either the cost-effectiveness or the cost-benefit of vaccination, and some found this strategy to be cost-saving [15]. Peasah et al. reported that 12 out of 18 studies focusing on the economic benefits of influenza vaccination of children found it to be cost-saving [16]. A recent systematic literature review included 10 studies focused on vaccination of school-aged children; they found vaccination of all children versus only high-risk children to be dominant to $47,000 per quality adjusted life year (QALY, societal) and to $18,000 per QALY (health care system) [17]. Unfortunately, most of these studies were focused on high-income countries, particularly the United States and selected European countries. Thus, further research is necessary to evaluate the impact of influenza vaccination in other regional contexts.

In this study we analysed epidemiologic and disease burden data to assess the clinical effects and economic benefits of expanding the current national influenza vaccination schedule of Mexico to include school-aged population (5 to 11 years). Using a static 1-year model that incorporates herd effect, we assessed the cost-effectiveness of such a change in policy, with the reduction in the number of influenza cases as the primary health outcome from the societal perspective.

## Methods

A cross-sectional epidemiological study was performed using the following databases: 1) a de-identified database from Mexico’s Influenza Surveillance System (SISVEFLU) obtained upon written request from Mexico’s General Directorate of Epidemiology, which included all influenza records from November 2009 to October 2018; 2) the mortality database of the National Mortality Epidemiological and Statistical System (SEED) for the period of 2010–2015 (data from 2009 and 2016–2018 were not available) [18], and 3) discharge and hospitalisation data obtained from the Automated Hospital Discharge System (SAEH) for the period 2010–2016 (data from 2009 and 2017–2018 were not available) (Additional file 1**: Supplement 1; Table S1.1**) [19]. Written approval was obtained from Mexico’s Ministry of Health to use these administrative data for academic purposes; written informed consent from patients was not required for this study. For both SEED and SAEH, codes from the International Statistical Classification of Diseases and Related Health Problems, 10th revision, (ICD-10) were used for the selection of cases; the codes used are listed in Additional file 1**: Supplement 1; Text S1.1**. Of note, Mexico began using ICD-10 codes in 1998 when their use was adopted into epidemiologic surveillance information systems. Data for projections of the Mexican population for the study period were obtained from the National Population Council database [20].

SISVEFLU uses a network of monitoring health care units across the country and has been automated since 2009. It is designed to provide timely and quality information on trends of circulating viral strains and the occurrence of severe cases [21]. Cases are initially classified as either ILI or severe acute respiratory infection (SARI). Diagnosis is confirmed by polymerase chain reaction (PCR) at FluNet collaborating facilities of the National Network of Public Health Laboratories (Red Nacional de Laboratorios de Salud Pública) [22].

For the purpose of this study, we used the case definitions for ILI and SARI used in the current Mexican influenza surveillance guidelines [22]. The school-aged population was defined as children between the ages of 5 years and 11 years, 11 months and 30 days.

For estimating the costs of influenza cases, the following seven scenarios were built, both for ambulatory patients (scenarios 1 through 3) and for inpatients (scenarios 4 through 7), based on data analysed from SISVEFLU:

- **Scenario 1:** Symptomatic individual visited an outpatient clinic, had a positive PCR result for influenza, was managed only in ambulatory care and had a complete recovery.
- **Scenario 2:** Symptomatic individual visited an outpatient clinic, had a positive PCR result for influenza for which, due to the severity, they were referred for hospital care where they had a complete recovery and were discharged.
- **Scenario 3:** Symptomatic individual visited an outpatient clinic, had a positive PCR result for influenza and, due to severity, was referred for hospital care and died.
- **Scenario 4:** Symptomatic individual visited a hospital ER, had a positive PCR result for influenza, was discharged to an outpatient clinic for follow-up and had a complete recovery.
- **Scenario 5:** Symptomatic individual visited a hospital ER, had a positive PCR result for influenza, was admitted to hospital for follow-up with non-severe clinical status and had a complete recovery.
- **Scenario 6:** Symptomatic individual visited a hospital ER, had a positive PCR result for influenza, was admitted for follow-up with severe clinical status and had a complete recovery.
- **Scenario 7:** Symptomatic individual visited a hospital ER, had a positive PCR result for influenza, was admitted for hospital follow-up and died.

**Fig. 1:**
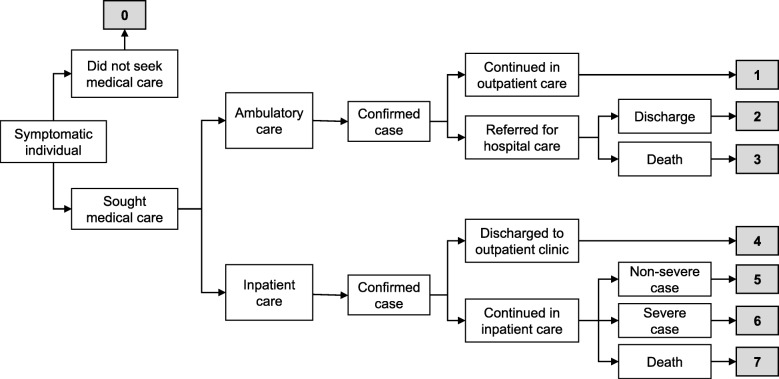
Decision tree for case classification

A scenario 0 was considered, in which symptomatic individuals did not request medical care and self-medicated with over-the-counter drugs. We assumed that the outcome in this scenario was complete recovery. Figure 1 shows the decision tree for case classification with all scenarios.

Assumptions for clinical management of cases for each scenario are summarised in Table 1 and were defined according to the national clinical guidelines for the prevention, diagnosis, and treatment of seasonal influenza in Mexico, as follows [23]:

**Table 1:** Clinical management by scenarios

	No medical care	Outpatient only	Hospitalisation: Referred from outpatient clinic		Hospitalisation: admitted through ER			
Scenario	0	1	2	3	4	5	6	7
Health outcome	Not demanding medical care	Outpatient only	Hospitalisation, discharge	Hospital care, death	Outpatient	Hospitalisation, non-severe	Hospitalisation, severe	Hospitalisation, death
**Diagnosis**								
Number of outpatient consultations		1	1	1				
Number of ER consultations					1	1	1	1
Percent of patients diagnosed using PCR (%)		10	10	10	100	100	100	100
**Direct costs**								
Number of outpatient consultations		1						
Number of specialist consultations			2	3	1	2	3	4
|Amantadine (Y/N)	Y	N	N	N	N	N	N	N
Oseltamivir (Y/N)	N	Y	Y	Y	Y	Y	Y	Y
Paracetamol (Y/N)	Y	Y	Y	Y	Y	Y	Y	Y
Bacteriologic culture (Y/N)	N	N	Y	Y	N	Y	Y	Y
Ceftriaxone (Y/N)	N	N	Y	Y	N	Y	Y	Y
Hospitalisation (days)			1	6.1	2	6.1	9.15	6.1
**Indirect costs**								
Medical disability (days)			3	14	14	14	24	
Years of life lost				X				X

Abbreviations: *ER* emergency room; *PCR* polymerase chain reaction

### Laboratory diagnosis

Influenza was confirmed using real-time PCR. According to the epidemiological surveillance guidelines [22], in primary health care units, sample collection for confirmation is only required for 10% of cases, whereas in secondary and tertiary health care units, 100% of cases are submitted for confirmation. In cases with suspicion of bacterial coinfection, throat swab culture is indicated.

### Medical consultations

Cases detected and managed in outpatient clinics (scenario 1) require a single medical consultation with no follow-up. Cases admitted to hospital via an outpatient clinic (scenario 2) require two medical consultations; the first for clinical diagnosis and the second at discharge with medical disability leave. For scenario 3 with admission to hospital via an outpatient clinic that results in death, the requirement is one initial consultation at the outpatient care facility and three subsequent consultations at the hospital. For hospital-managed cases admitted through the ER (scenarios 4 through 7), an emergency consultation was required. Cases admitted for hospitalisation had one, two, three, or four medical consultations in scenarios 4, 5, 6, and 7 respectively, assuming a proportional increase in the number of medical consultations, consistent with the severity of the case.

### Drugs

For individuals not requesting medical care (scenario 0) and only requiring over-the-counter drugs, amantadine use was assumed. For all confirmed cases, patients were assumed to have been prescribed oseltamivir for influenza treatment and paracetamol for acute pain management. For cases with a bacterial coinfection requiring antibiotic treatment, use of ceftriaxone was assumed.

### Days of hospitalisation

Patients admitted via an outpatient clinic who were referred for hospitalisation and later discharged were assumed to have had a 1-day hospital stay. Patients admitted via an outpatient clinic who were referred for hospitalisation that resulted in death were considered to have had a 6.1-day hospital stay based on results obtained from the SAEH database [19]: this assumption takes into consideration that hospital care is provided independently of the admitting area or laboratory confirmation of the case. Patients admitted to hospital for observation via the ER who were discharged for follow-up at an outpatient clinic were considered to have had a 2-day hospital stay. Patients admitted via the ER for medical care who were classified as non-severe were considered to have had a 6.1-day hospital stay. Patients admitted to hospital via the ER who were classified as severe were considered to have had 50% longer hospital stays (9.15 days) than patients with non-severe cases. A 6.1-day stay was assumed for patients admitted via the ER whose outcome was death.

### Days of medical disability leave

For patients diagnosed in outpatient clinics who did not require admission to hospital (scenario 1), a 3-day medical leave was assumed. For patients diagnosed in outpatient clinics who were referred to hospital (scenario 2) and those admitted via the ER and hospitalised as non-severe cases (scenario 5), a 7-day medical leave was assumed after hospital discharge, for a total of 13 days of absence. For patients admitted via the ER and hospitalised as severe cases (scenario 6), a 14-day medical leave was assumed after hospital discharge, for a total of 23 days of absence [24].

It was assumed that school-aged population with influenza was monitored by an adult caregiver (parent or close relative) for the duration of their sickness, which was assumed to have had an effect on the productivity of the adult caregiver.

### Years of life lost (YLL)

For the YLL calculation, the age of each influenza-confirmed death that was registered in SISVEFLU was individually considered. The lower and upper limits were as follows: lower limit, age of 1 year for all individuals; upper limit, age of 73 or 78 years for males and females, respectively [25, 26]. The upper age limit was determined according to the current life expectancy in Mexico; these ages were later weighed according to the population distribution by sex.

### National estimates of influenza cases

Given that SISVEFLU cases were recorded from monitoring facilities and the data are not defined as sentinel, data cannot be extrapolated directly to population-wide estimates (Additional file 1**: Supplement 1; Text S1.2, Fig. S1**). Therefore, the total number of influenza cases in Mexico were estimated by indirectly standardising reported values of influenza incidence in the United States for each season and age group [27] into the Mexican population structure according to official population projections (Additional file 1**: Supplement 1; Tables S1.2, S1.3, S1.4, S1.5**) [20].

National estimated cases were then allocated into the different scenarios, considering 1) the probability of not demanding medical care (scenario 0), as reported by Molinari et al. for different age groups [28], 2) where cases were diagnosed according to the SISVEFLU database (outpatient clinic or hospital), and 3) likelihood of occurrence of health outcome (ambulatory discharge, hospitalisation and subsequent discharge, or death). Further details of the method used to estimate national cases are available in Additional file 1**: Supplement 2, Text S2, Tables S2.1–S2.9; and** Fig. 1.

### Unit costs for the estimation of economic burden of influenza

Public costs were used for the estimation of direct medical care costs by each of the institutions that comprise the Mexican Health System and weighed by the proportion of the population affiliated in each institution for the influenza seasons from 2009 to 2010 to 2018–2019 (Additional file 1**: Supplement 3; Tables S3.1, S3.2, S3.3, S3.4, S3.5**). The productivity loss associated with a working day lost was valued at the average daily wage of an individual (obtained from the 2018 National Survey of Household Income and Expenditure); costs associated with premature deaths were projected using the WHO’s recommended 5% discount rate.

Costs were originally obtained in Mexican pesos (MXN) and later converted to 2018 constant prices using the National Consumer Price Index published by Mexico’s National Bureau of Statics and Geography (Additional file 1**: Supplement 3; Table S3.6**). Data are presented in US dollars (USD) considering the average exchange rate published in the Official Federal Gazette (USD 1 = MXN 19.2155).

### Costs of vaccination

The price per dose of influenza vaccine was obtained from Mexico’s Ministry of Health for 2018 (MXN 56.0, USD 2.91); the cost of administration was obtained from Gutierrez and Bertozzi’s study and converted to 2018 prices [29]. The cost of transportation and storage was considered as a percentage of the price per dose, considering that the implementation of influenza vaccination in school-aged population would take place at primary schools.

### Vaccine coverage and effectiveness

Effectiveness of the influenza vaccine was set at 50%, which is the average effectiveness of the influenza vaccine in the Northern Hemisphere of the Americas as published elsewhere (Additional file 1**: Supplement 4; Table S4**) from influenza seasons from 2009 to 2010 to 2017–2018. Coverage was conservatively defined at 50%, assuming slow adoption rates for school-based influenza immunisations in the first years of implementation.

To account for herd effect, we followed the methodology of Van Vlaenderen et al., who fitted a linear function to point estimates from previous published studies that incorporated herd effect due to vaccinating children against seasonal influenza for both school-aged population and the rest of the population [30]. Eqs. 1 and 2, shown below, were used to estimate the herd effect for the two populations:

Equation 1

$$ {RR}_{unvaccinated\_ childre\mathrm{n}}=1-\alpha \ast effective\ coverage\ in\ children, $$

where *α* represents the conservative (*α* = 1) parameter to capture the herd effect, and

$$ effective\ coverage\ in\ children= vaccination\ coverage\ast vaccine\ efficacy. $$

Equation 2

$$ {RR}_{other\_ age\_ groups}=1-\beta \ast effective\ coverage\ in\ children\ast {P}_{children}, $$

where *β* represents the conservative (*β* = 1) parameter to capture the herd effect, *P*_*children*_ refers to the proportion of children vaccinated, and

$$ effective\ coverage\ in\ children= vaccination\ coverage\ast vaccine\ efficacy. $$

The parameter used to account for herd effect was the proportion of the school-aged population that need to be vaccinated to achieve a relative risk (RR) of 0 (zero risk of infection). Two parameters, an optimistic one and a conservative one, were defined for the school-aged population (optimistic, 훼=1.2031 [83.1%]; conservative, 훼=1 [100%]) and the rest of the population (optimistic, 훼=4.6656 [21.4%]; conservative, 훼=1 [100%]); the conservative parameter was used in this study.

Disaggregated data by age group enabled estimation of the herd effect, particularly in those not vaccinated under the current schedule (Additional file 1**: Supplement 2; Table S2.4**).

### Influenza outcomes

The primary health outcome for this study was influenza cases avoided, which in turn led to secondary health outcomes such as reductions in outpatient consultations, lost working days, hospitalisations, and deaths.

### Sensitivity analysis

We performed a sensitivity analysis to assess whether the cost-effectiveness of immunising the school-aged population was sustained when using either a conservative or optimistic scenario, considering changes to the base case scenario in both vaccination coverage and vaccination effectiveness. In the conservative scenario, we assumed a vaccination coverage of 30% and a vaccination effectiveness of 19%, which is the lowest effectiveness reported in any influenza vaccine that was used from season 2009–2010 through season 2017–2018 in the Northern Hemisphere of the Americas. For the optimistic scenario we assumed a vaccination coverage of 70% and a vaccination effectiveness of 68%, which was the highest effectiveness reported in the same period and region (Additional file 1**: Supplement 4; Table S.4**).

## Results

### Epidemiology

From November 2009 to October 2018, Mexico’s SISVEFLU system recorded 50,900 laboratory confirmed cases out of 390,862 probable ILI/SARI cases of all ages (13.02% positivity proportion). The number of probable ILI/SARI cases in the school-aged population (5–11 years) was 32,232, of which 5121 (15.89%) were confirmed (Table 2).

**Table 2:** Number of confirmed cases and deaths in the school-aged population

Season^**a**^	Confirmed cases									Deaths								
	ILI			SARI			Total			ILI			SARI			Total		
	Influenza type			Influenza type			Influenza type			Influenza type			Influenza type			Influenza type		
	A	B	Total	A	B	Total	A	B	Total	A	B	Total	A	B	Total	A	B	Total
**2009–2010**^**b**^	151	5	**156**	103	0	**103**	254	5	**259**	1	0	**1**	3	0	**3**	4	0	**4**
**2010–2011**	274	99	**373**	204	12	**216**	478	111	**589**	0	0	**0**	0	0	**0**	0	0	**0**
**2011–2012**	490	38	**528**	223	5	**228**	713	43	**756**	1	0	**1**	6	0	**6**	7	0	**7**
**2012–2013**	152	207	**359**	55	40	**95**	207	247	**454**	0	0	**0**	1	1	**2**	1	1	**2**
**2013–2014**	304	75	**379**	237	39	**276**	541	114	**655**	1	1	**2**	15	0	**15**	16	1	**17**
**2014–2015**	123	69	**192**	75	42	**117**	198	111	**309**	0	0	**0**	1	2	**3**	1	2	**3**
**2015–2016**	363	208	**571**	258	102	**360**	621	310	**931**	8	2	**10**	14	0	**14**	22	2	**24**
**2016–2017**	228	189	**417**	187	56	**243**	415	245	**660**	3	0	**3**	6	1	**7**	9	1	**10**
**2017–2018**	229	138	**367**	70	29	**99**	299	167	**466**	2	1	**3**	5	0	**5**	7	1	**8**
**2018–2019**^**c**^	19	8	**27**	12	3	**15**	31	11	**42**	0	0	**0**	0	0	**0**	0	0	**0**
**TOTAL**	**2333**	**1036**	**3369**	**1424**	**328**	**1752**	**3757**	**1364**	**5121**	**16**	**4**	**20**	**51**	**4**	**55**	**67**	**8**	**75**

^a^A season was defined as beginning in epidemiological week 34 of year 1 (mid-August), and ending in epidemiological week 33 (early August) of year 2

^b^The 2009–2010 season includes records starting from November 2009

^c^The 2018–2019 season includes only records from August to October 2018

The data used in this table were obtained from SISVEFLU

Abbreviations: *ILI* influenza-like illness; *SARI* severe acute respiratory infection; *SISVEFLU* Mexico’s Influenza Surveillance System

Influenza A virus was isolated in 73.3% of the cases. This viral serotype accounted for 89.3% of deaths. The season with the highest record of confirmed cases was 2015–2016, with 621 influenza A cases and 310 influenza B cases reported. Regarding clinical presentation, as expected, most of the confirmed cases were classified as ILI (65.8%), whereas most deaths occurred in the SARI classification (73.3%). Results for cases, clinical presentation, viral types, and deaths were also consistent between seasons.

As for lethality (Table 3), the overall value was 1.46% (75/5121). Nonetheless, there are important variations depending on the infecting virus. The highest lethality was recorded for A H1N1 (2.82%), followed by B Yamagata (0.71%), B undetermined lineage (0.71%), and A H3N2 (0.68%). No deaths were recorded for B Victoria or influenza A not subtyped.

**Table 3:** Cases, deaths, and lethality in the school-aged population by season

Influenza type	Subtype	Indicator	2009–2010	2010–2011	2011–2012	2012–2013	2013–2014	2014–2015	2015–2016	2016–2017	2017–2018	2018–2019	TOTAL
**A**	**H1N1**	Cases	212	12	663	33	412	3	328	267	97	30	2057
		Deaths	4	0	7	0	16	0	18	8	5	0	58
		Lethality (%)	1.89	0.00	1.06	0.00	3.88	0.00	5.49	3.00	5.15	0.00	2.82
	**H3N2**	Cases	8	230	18	160	114	187	287	138	184	1	1327
		Deaths	0	0	0	1	0	1	4	1	2	0	9
		Lethality (%)	0.00	0.00	0.00	0.63	0.00	0.53	1.39	0.72	1.09	0.00	0.68
	**Not** **subtyped**	Cases	34	236	32	14	15	8	6	10	18	0	373
		Deaths	0	0	0	0	0	0	0	0	0	0	0
		Lethality (%)	0.00	0.00	0.00	0.00	0.00	0.00	0.00	0.00	0.00	0.00	0.00
	**Total A**	Cases	254	478	713	207	541	198	621	415	299	31	3757
		Deaths	4	0	7	1	16	1	22	9	7	0	67
		Lethality (%)	1.57	0.00	0.98	0.48	2.96	0.51	3.54	2.17	2.34	0.00	1.78
**B**	**Victoria**	Cases	0	0	10	58	37	12	49	16	56	3	241
		Deaths	0	0	0	0	0	0	0	0	0	0	0
		Lethality (%)	0.00	0.00	0.00	0.00	0.00	0.00	0.00	0.00	0.00	0.00	0.00
	**Yamagata**	Cases	0	0	1	54	6	40	63	80	33	3	280
		Deaths	0	0	0	0	0	1	0	1	0	0	2
		Lethality (%)	0.00	0.00	0.00	0.00	0.00	2.50	0.00	1.25	0.00	0.00	0.71
	**Undetermined lineage**	Cases	5	111	32	135	71	59	198	149	78	5	843
		Deaths	0	0	0	1	1	1	2	0	1	0	6
		Lethality (%)	0.00	0.00	0.00	0.74	1.41	1.69	1.01	0.00	1.28	0.00	0.71
	**Total B**	Cases	5	111	43	247	114	111	310	245	167	11	1364
		Deaths	0	0	0	1	1	2	2	1	1	0	8
		Lethality (%)	0.00	0.00	0.00	0.40	0.88	1.80	0.65	0.41	0.60	0.00	0.59
**Total influenza**		Cases	259	589	756	454	655	309	931	660	466	42	5121
		Deaths	4	0	7	2	17	3	24	10	8	0	75
		Lethality (%)	1.54	0.00	0.93	0.44	2.60	0.97	2.58	1.52	1.72	0.00	1.46

The data used in this table were obtained from Mexico’s influenza surveillance system, SISVEFLU

The highest lethality was recorded in children aged 5 and 11 years with influenza A H1N1 (3.33 and 3.36%, respectively) (Table 4).

**Table 4:** Cases, deaths, and lethality in the school-aged population by age and influenza type

Influenza type	Subtype	Indicator	Age							Total
			5	6	7	8	9	10	11	
**A**	**H1N1**	Cases	360	381	332	300	229	217	238	**2057**
		Deaths	12	12	10	6	5	5	8	**58**
		Lethality (%)	3.33	3.15	3.01	2.00	2.18	2.30	3.36	**2.82**
	**H3N2**	Cases	224	234	225	170	170	164	140	**1327**
		Deaths	3	3	1	1	1	0	0	**9**
		Lethality (%)	1.34	1.28	0.44	0.59	0.59	0.00	0.00	**0.68**
	**Not subtyped**	Cases	80	58	72	45	48	38	32	**373**
		Deaths	0	0	0	0	0	0	0	**0**
		Lethality (%)	0.00	0.00	0.00	0.00	0.00	0.00	0.00	**0.00**
	**Total A**	Cases	664	673	629	515	447	419	410	**3757**
		Deaths	15	15	11	7	6	5	8	**67**
		Lethality (%)	2.26	2.23	1.75	1.36	1.34	1.19	1.95	**1.78**
**B**	**Victoria**	Cases	50	34	31	47	34	22	23	**241**
		Deaths	0	0	0	0	0	0	0	**2**
		Lethality (%)	0.00	0.00	0.00	0.00	0.00	0.00	0.00	**0.83**
	**Yamagata**	Cases	39	41	39	57	38	38	28	**280**
		Deaths	0	0	0	0	0	2	0	**0**
		Lethality (%)	0.00	0.00	0.00	0.00	0.00	5.26	0.00	**0.00**
	**Undetermined lineage**	Cases	127	153	144	129	114	80	96	**843**
		Deaths	2	1	0	0	2	1	0	**6**
		Lethality (%)	1.57	0.65	0.00	0.00	1.75	1.25	0.00	**0.71**
	**Total B**	Cases	216	228	214	233	186	140	147	**1364**
		Deaths	2	1	0	0	2	3	0	**8**
		Lethality (%)	0.93	0.44	0.00	0.00	1.08	2.14	0.00	**0.59**
**Total influenza**		Cases	880	901	843	748	633	559	557	**5121**
		Deaths	17	16	11	7	8	8	8	**75**
		Lethality (%)	1.93	1.78	1.30	0.94	1.26	1.43	1.44	**1.46**

The data used in this table were obtained from Mexico’s influenza surveillance system, SISVEFLU

**Fig. 2:**
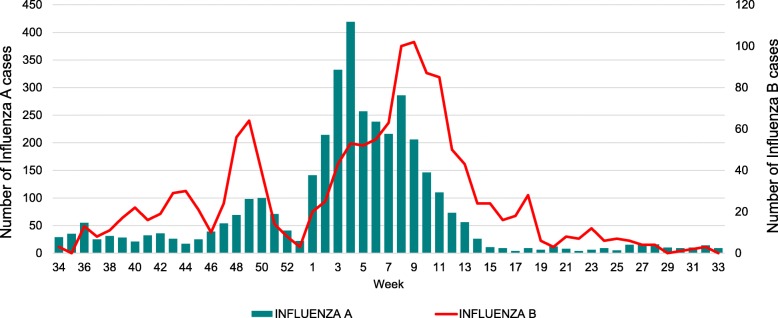
Influenza A and B confirmed cases in the school-aged population by epidemiological week (seasons 2009–2018). The data used in this figure were obtained from Mexico’s influenza surveillance system, SISVEFLU

When analysing data from the complete study period, influenza cases peaked between epidemiological weeks 4 and 9. However, in school-aged population in Mexico, influenza B transmission seemed to have a longer duration (started earlier and ended later) in comparison to the duration of influenza A in the same season (Fig. 2).

### Incidence

Table 5 shows the cumulative incidence of influenza in school-aged children by age and season.

**Table 5:** Incidence of influenza per 100,000 inhabitants in school-aged population by age and season

Age	Seasons ^**a**^								Average
	2010–2011	2011–2012	2012–2013	2013–2014	2014–2015	2015–2016	2016–2017	2017–2018	
**5**	5.32	4.09	2.99	4.78	2.60	8.18	5.59	3.70	**4.66**
**6**	4.30	5.72	2.94	5.36	2.60	7.99	5.45	4.10	**4.81**
**7**	4.98	5.90	3.24	3.97	2.28	6.00	5.71	3.15	**4.40**
**8**	3.12	4.58	2.97	4.89	1.92	6.22	4.22	3.46	**3.92**
**9**	3.08	4.24	2.94	3.82	1.25	5.00	2.60	3.37	**3.29**
**10**	2.86	4.11	2.86	2.58	1.96	4.68	2.77	1.79	**2.95**
**11**	2.56	5.01	2.28	3.80	1.20	3.60	3.30	1.39	**2.89**

^**a**^The 2009–2010 and 2018–2019 seasons were not analysed, since the data were not complete

The data used in this table were obtained from Mexico’s influenza surveillance system, SISVEFLU

These data show that the cumulative incidence of influenza had a tendency to decrease with age. The highest incidence was reported amongst children aged 5 and 6 years.

### Hospital discharges

**Fig. 3:**
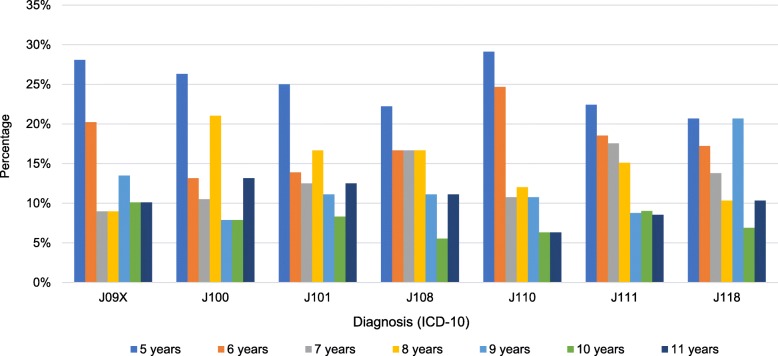
Discharges by age and principal diagnosis in the school-aged population (*n* = 814) Diagnosis codes: J09X, influenza due to certain identified influenza virus; J100, influenza with pneumonia, other influenza virus identified; J101, influenza with other respiratory manifestations, other influenza virus identified; J108, influenza with other manifestations, other influenza virus identified; J110, influenza with pneumonia, virus not identified; J111, influenza with other respiratory manifestations, virus not identified; J118, influenza with other manifestations, virus not identified. The data used in this figure were obtained from the Automated Hospital Discharge System, SAEH [19]. Abbreviation: ICD-10: International Classification of Diseases, 10th revision

Table 6 and Fig. 3 show total hospital discharges for the 2010–2016 period, in which influenza was the main diagnosis.

**Table 6:** Hospital discharges with influenza as the main diagnosis in school-aged population by ICD-10 (2010–2016)

ICD-10	Main diagnosis	Discharges	Total bed-days	Average bed-days
J09X	Influenza due to certain identified influenza virus	89	561	6.3
J100	Influenza with pneumonia, other influenza virus identified	38	290	7.6
J101	Influenza with other respiratory manifestations, other influenza virus identified	72	272	3.8
J108	Influenza with other manifestations, other influenza virus identified	18	78	4.3
J110	Influenza with pneumonia, virus not identified	158	936	5.9
J111	Influenza with other respiratory manifestations, virus not identified	410	1370	3.3
J118	Influenza with other manifestations, virus not identified	29	89	3.1
**Total**		**814**	**3596**	**4.4**

The data used in this table were obtained from the Automated Hospital Discharge System, SAEH [19]

Abbreviation: *ICD-10* International Statistical Classification of Diseases and Related Health Problems, 10th revision

There were 814 discharges from hospital of school-aged children during the study period, accounting for 3596 bed-days, with an average of 4.4 bed-days per child. The most frequent diagnosis was influenza with other respiratory manifestations, virus not identified (J111), followed by influenza with pneumonia, virus not identified (J110).

### Mortality

Table 7 shows mortality by season for all age groups. Overall mortality was low (0.3/100,000 individuals), however, it was remarkably higher in the youngest and older age groups. For school-aged population, mortality due to influenza was also low.

**Table 7:** Mortality by age group and season (per 100,000 inhabitants)

Age group	Season^**a**^					Average mortality
	2010–2011	2011–2012	2012–2013	2013–2014	2014–2015	
**< 1**	0.8	1.3	1.0	1.2	0.4	0.9
**1–4**	0.1	0.1	0.4	0.2	0.1	0.2
**5–11**	0.0	0.0	0.0	0.1	0.0	0.0
**12–17**	0.1	0.0	0.0	0.1	0.0	0.0
**18–49**	0.1	0.2	0.0	0.7	0.0	0.2
**50–59**	0.8	0.9	0.0	2.0	0.1	0.8
**≥60**	1.3	1.0	0.1	2.0	0.3	0.9
**Total**	**0.2**	**0.3**	**0.1**	**0.8**	**0.1**	**0.3**

^**a**^The 2009–2010 and 2015–2016 seasons were not analysed, since the data were not complete

The data used in this table were obtained from the National Death Epidemiological and Statistics Subsystem, SEED [18] and the National Population Council, CONAPO [20]

### Cost-effectiveness

The estimated expansion of the influenza immunisation programme to the school-aged population over the 2018–2019 season, with an assumed 50% coverage and 50% effectiveness, resulted in 671,461 cases avoided. This was associated with 262,800 fewer outpatient consultations; 154,100 fewer ER consultations; 97,600 fewer hospitalisations, and 15 fewer deaths (Table 8). The decrease in demand for the aforementioned services would also reduce the pressure on the public health system.

**Table 8:** Estimated influenza-related events avoided by immunising school-aged population

Outcomes	No influenza immunisation	Influenza immunisation	Avoided
Influenza cases	11,165,666	10,494,205	671,461
Outpatient consultations	4,166,168	3,903,356	262,812
Emergency room consultations	3,091,393	2,937,250	154,143
Hospitalisations	1,962,486	1,864,850	97,637
Deaths	414	399	15

When looking at the estimated number of cases avoided by age-group (Table 9), 55.42% of the cases avoided were from school-aged population. In terms of demand for influenza-related health services, the vaccination had the largest effect in school-aged population. In addition, it is worth noting the effect of vaccinating school-aged children on the reduction in the demand of influenza-related health services by population over 60 years old, who consequently required fewer hospitalisation services.

**Table 9:** Influenza-related events avoided by immunising the school-aged population by age group

Outcomes	Age group					Total
	0–4 years	5–11 years	12–49 years	50–59 years	≥60 years	
Influenza cases	46,055	372,099	169,692	46,237	37,380	671,461
	6.86%	55.42%	25.27%	6.89%	5.56%	
Outpatient consultations	16,520	152,847	68,997	14,593	9856	262,812
	6.29%	58.16%	26.25%	5.55%	3.75%	
Emergency room consultations	21,237	66,775	30,353	12,812	22,967	154,143
	13.78%	43.32%	19.69%	8.31%	14.90%	
Hospitalisations	13,352	42,143	19,420	8138	14,585	97,637
	13.67%	43.16%	19.89%	8.34%	14.94%	
Deaths	1	2	5	3	4	15
	6.67%	13.33%	33.33%	20.00%	26.67%	

The number of estimated cases avoided represented a decrease in the economic burden for the Mexican health care system of approximately 112 million USD, as shown in Table 10. Of these costs avoided, 93.5 million USD (83.5%) was from hospitalisations, 14.6 million USD (13.0%) was from medical consultations, and 14.4 million USD (12.9%) was from productivity loss. Direct costs accounted for 86.27% and indirect costs accounted for 13.73% of the total costs avoided. The costs avoided from hospitalisations and the sum of the costs avoided from medical consultations and productivity loss were 3.41 and 1.06 times the cost of the immunisation programme in the school-aged population, respectively; thus demonstrating that the immunisation programme was a cost-saving strategy.

**Table 10:** Total economic benefits of influenza immunisation to the school-age population

Influenza-associated costs	No influenza immunisation	Influenza immunisation	Costs avoided
Direct costs			
Laboratory diagnosis	259,878,840	246,608,193	13,270,647
Medical consultations	266,216,669	251,638,144	14,578,525
Drugs	43,978,880	41,388,986	2,589,893
Hospitalisations	1,866,950,845	1,773,439,651	93,511,195
Influenza immunisation to school-aged population		27,421,602	−27,421,602
**Total direct costs (third-party payer perspective)**	2,437,025,233	2,340,496,577	96,528,657
Indirect costs			
Productivity loss	267,469,325	253,037,896	14,431,429
Premature death	29,368,240	28,338,858	1,029,382
Total indirect costs	296,837,565	281,376,755	15,460,811
**Total costs of influenza (societal perspective)**	**2,733,862,799**	**2,621,873,331**	**111,989,467**

All costs are in USD

Costs avoided (Table 11) were highest for the school-aged population (35.16 million USD), followed by the 12–49 year old population. The costs of hospitalisations in people over 60 years old (13.8 million USD) represented 76.69% of total costs avoided for this age group. This study was conservative and did not include additional costs due to complications from comorbidities; it is likely that the costs avoided in this age group are greater.

**Table 11:** Costs avoided by age group

Influenza-associated costs	Age group				
	0–4 years	5–11 years	12–49 years	50–59 years	≥60 years
Direct costs					
Laboratory diagnosis	1,705,270	5,970,134	2,729,797	1,060,393	1,805,053
Medical consultations	1,537,700	7,238,955	3,285,458	1,040,038	1,476,373
Drugs	200,447	1,403,033	638,823	177,367	170,223
Hospitalisations	12,948,383	40,807,242	18,282,368	7,697,330	13,775,872
Influenza immunisation to school-aged population		−27,421,602			
**Total direct costs avoided (third-party payer perspective)**	16,391,799	27,997,763	24,936,446	9,975,128	17,227,521
Indirect costs					
Productivity loss	2,243,927	7,026,866	3,186,588	1,357,530	616,517
Premature death	16,215	137,958	526,445	229,548	119,216
Total indirect costs avoided	2,260,142	7,164,824	3,713,033	1,587,079	735,733
**Total costs of influenza avoided (societal perspective)**	**18,651,941**	**35,162,586**	**28,649,479**	**11,562,206**	**17,963,254**

All costs are in USD

### Sensitivity analysis

Table 12 shows the results of estimated influenza-related events avoided in the three scenarios (base case, conservative, and optimistic). Reducing influenza vaccine coverage and effectiveness to 30 and 19% (conservative), respectively, resulted in 153,000 avoided cases of influenza which were associated with 95,000 consultations (outpatient and emergency room), 22,200 hospitalisations, and three deaths. The optimistic scenario used estimations of 70 and 68% by increasing vaccination coverage and effectiveness from the base case estimations by a respective 20 and 18%. This resulted in an estimated 1,270,000 avoided cases of influenza, which was an increase of 1.9 times the number of cases avoided in the base case scenario.

**Table 12:** Estimated influenza-related events avoided by immunising the school-aged population, sensitivity analysis

Events avoided	Base case	Conservative	Optimistic
Vaccination coverage	50%	30%	70%
Vaccine effectiveness	50%	19%	68%
Influenza cases	671,461	153,093	1,278,462
Outpatient consultations	262,812	59,921	500,395
Emergency Room consultations	154,143	35,145	293,489
Hospitalisations	97,637	22,261	185,900
Deaths	15	3	28

Using the aforementioned values for the number of influenza-related events avoided, we assessed the economic benefits of the three scenarios. The results are shown in Table 13. In the conservative scenario, influenza immunisation of the school-aged population resulted in a savings of 15.3 million USD, of which 11.8 million USD was considered direct savings; the remaining 3.5 million USD was considered indirect savings. This analysis demonstrates that even when using a conservative scenario, immunising the school-aged population is a cost-saving intervention and should be considered a beneficial strategy considering the current policy of not vaccinating this population.

**Table 13:** Total economic benefits of influenza immunisation in the school-age population, sensitivity analysis

Influenza-associated costs	Base case	Conservative	Optimistic
Vaccination coverage	50%	30%	70%
Vaccine effectiveness	50%	19%	68%
Direct Costs			
Laboratory diagnosis	−13,270,647	−3,025,707	−25,267,311
Medical consultations	−14,578,525	−3,323,904	−27,757,511
Drugs	−2,589,893	−590,496	−4,931,157
Hospitalisations	−93,511,195	−21,320,552	− 178,045,314
Influenza immunisation of the school-aged population	27,421,602	16,452,961	38,390,243
Total direct costs (Third-Party Payer perspective)	−96,528,657	−11,807,698	− 197,611,050
Indirect costs			
Productivity loss	−14,431,429	−3,290,366	−27,477,440
Premature death	−1,029,382	−234,699	−1,959,943
Total indirect costs	−15,460,811	−3,525,065	−29,437,384
**Total cost of influenza**	−111,989,467	−15,332,762	−227,048,433

**Fig. 4:**
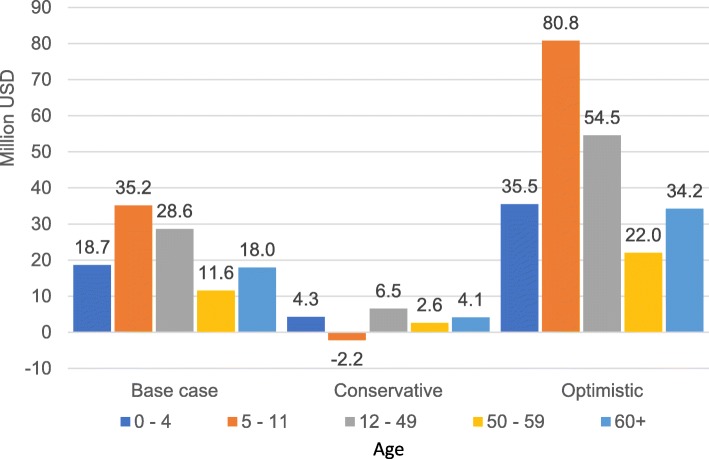
Costs avoided by influenza immunisation of the school-aged population (by age group)

Figure 4 shows costs avoided by age group in each of the three scenarios. In both the base case and the optimistic scenario, the greatest savings are seen in the school-aged population, where the intervention would take place. This is true even when the costs of the intervention itself are taken into account (27.4 million USD [base case]; 38.4 million USD [optimistic]). In the conservative scenario, the costs of the intervention surpassed savings from cases avoided (2.2 million USD) in the school-aged population; however, these costs are more than offset by savings from cases avoided (17.5 million USD) in other age groups due to the herd effect.

## Discussion

Children and adolescents (5 to 19 years) play an important role in the spread of influenza in the community and are considered the main disseminators of influenza or “superspreaders” [31]. This is because they have less acquired immunity [32], a longer period of virus-shedding once infected [33], and a higher number of contacts with other people once they acquire the disease [34].

The population between the ages of 5 and 11 years (school-aged) is highly relevant in regard to public health for a number of reasons, including their status as a potentially captive segment, specifically in the educational setting; intervention can be achieved in a swift and efficient manner. In the case of Mexico’s Immunisation Programme, this feature has allowed for achievement of adequate coverage for a number of vaccines. Nonetheless, this population is not considered a target group for influenza vaccination in the vast majority of countries, including Mexico.

Mexico’s school-aged population currently accounts for 12% of the total population. Demographic projections show that this group is due to diminish to approximately 10.5% in the next 30 years, representing around 14.6 million people (Additional file 1**: Supplement 4; Fig. S2**). Given the “superspreader” status of this age group, specific and effective public health strategies are required.

Our estimations showed that every year in Mexico there were an average of 10.8 million influenza cases, with an overall incidence rate of around 8.9% that is consistent with published literature [35]. The school-aged population identified in the surveillance system accounted for 10.06% of the cases (Additional file 1**: Supplement 5; Table S5.1**). Our estimation suggests that each year there are between 0.5 to 2.1 million influenza cases in this population (Additional file 1**: Supplement 5; Table S5.2**). Compared to other age groups, school-aged population has the second lowest incidence, lethality for influenza, and mortality (Additional file 1**: Supplement 5; Tables S5.3, S5.4, S5.5, S5.6**).

We speculate that there are two possible explanations for the relatively low burden of disease and the age-dependent tendencies observed. First, low lethality and mortality in this population may indicate a relatively benign clinical presentation, which may in turn trigger the resolution of the majority of cases in primary health care clinics, leaving the relatively small fraction of severe cases to be recorded in the SISVEFLU system via monitoring units (mostly hospitals). A non-excluding explanation may be a cohort effect favoured by either the exposure of this age group to the pandemic A H1N1 virus during the 2009–2010 season, the post-pandemic vaccination coverage, or multiple yearly influenza vaccinations in the last decade, which, as some studies suggest, may have provided effective protection [36–39]. Pandemic-induced immunity was previously shown to have a protective effect in children [36] and prior vaccination has been reported to modify vaccine effectiveness by providing some residual protection [39]. The serologic data required to test such hypotheses for the current study are not available.

Previous studies have recognised the role of the child population as key spreaders of influenza and that schools, day-care centres, and other places where children congregate play an important role in spreading influenza [34, 40, 41]. Although high mortality is not usually reported for this group [42], studies have recommended the inclusion of this population as a target group for influenza vaccination based on epidemiological parameters such as the Number Needed to Vaccinate or number of medical consultations [43].

Vaccination in the school-aged population has been shown to have both direct and indirect positive effects. Using a school-based vaccination approach has resulted in decreased influenza rates and improved school attendance [44]. In addition, it has been shown to reduce hospitalisations [45] and paediatric deaths [37]. As for indirect effects, medically attended acute respiratory illness (MAARI)-related ER visits during the Intense Influenza Outbreak Period (IIOP) decreased as vaccination rates increased for all populations [46].

Despite the potential benefits, it is important to consider the long-term effects of introducing influenza vaccination in a select population, as migration of the burden of disease to other age groups may occur. This situation may pose difficulties for public health, especially if members of the affected population present an increased frequency of risk factors that may modify morbidity and mortality [47]. This may explain the MAARI-related increase in hospitalisations during the IIOP (4% in adults aged > 50 years for every 20% increase in vaccination rates) [46].

In order to avoid negative effects and encourage positive effects in regard to averting influenza cases and influenza-related deaths [7], universal influenza vaccine coverage is considered best practice. However, universal vaccine coverage remains a challenging goal for most countries. A suitable alternative may be to plan a step-wise introduction of influenza vaccination in the young, such as primary school-aged population, thus gradually generating immunised cohorts while advancing the introduction of influenza vaccination to older groups until adequate coverage to effectively hinder influenza transmission is reached.

To our knowledge, this is one of the first studies to estimate the national impact of influenza immunisation of the school-aged population in a middle-income country. While Peasah et al. reviewed the cost and cost-effectiveness of influenza vaccination, very few of the studies reported national estimates, and of those, all but one (Thailand) were from high-income countries [16]. Jamotte et al. estimated the public health impact and economic benefit of introducing a quadrivalent vaccine as opposed to a trivalent vaccine, which was standard practice at the time of the study in Brazil, Colombia, and Panama [48]. However, their analysis did not include the school-aged population as it was not policy to vaccinate that age group at the time the study was conducted. The data reported in the present study demonstrated that the expansion of the current national immunisation programme to the school-aged population would greatly benefit the Mexican public health system, and is of value to health care policy makers in middle-income countries. Our analysis revealed that even when using a conservative scenario (30% vaccine coverage, 19% vaccine effectiveness), this intervention was still cost-effective. We believe there are two main reasons for this. Firstly, the cost of the influenza vaccine is significantly less expensive than the cost of treatment; the cost to vaccinate a child is approximately 3 USD, while the cost of treatment (including indirect costs) could be as high as 50,000 USD. Secondly, vaccinating children provides a significant herd effect. As previously mentioned, children are considered superspreaders; thus, vaccination of the school-aged population provides clinical benefits to other age groups by reducing influenza transmission. As noted in the sensitivity analysis (Fig. 4), although the cost of intervention was greater than the economic benefit in the conservative scenario, when the effect on the number of cases from other age groups was considered, the cost was offset, providing an overall economic benefit.

We speculate that the main reason the school-aged population is excluded from the current influenza vaccination guidelines in Mexico is because of a lack of reliable analyses to identify the necessity and potential benefits of expanding current target vaccination groups to include children. The World Health Organization’s Strategic Advisory Group of Experts’ (SAGE) 2012 paper on influenza immunization does not recommend the school-aged population as a target for vaccination. However, a new SAGE group was established in December 2017 to review scientific evidence to assess whether a recommendation for vaccinating the school-aged population should be supported [49]. We believe that the present manuscript provides such information, and that the data presented herein can facilitate the discussion towards updating the recommendations, particularly for middle-income countries.

Our study had several limitations. The databases used in this study did not completely overlap in duration which may have affected our results. Additionally, data regarding the number of school-aged children included in our study who received the influenza vaccination before the age of 5 years were not available. It has been reported that vaccination of children < 5 years of age provides benefit [50], so our inability to factor this variable into our analysis may have affected our results. Most of the monitoring health units included in the SISVEFLU database are public, and some bias may have been introduced by not including most private health care facilities. However, the economic analysis was made using national estimates and costs estimated from the three largest public health providers, which cover > 90% of the population in Mexico. Out-of-pocket expenses other than the cost of the over-the-counter drug, amantadine, were not considered when making assumptions regarding the economic impact of influenza. This was done in an effort to be conservative and not overestimate the economic benefits of the influenza vaccine; in doing so, we may have under-estimated such benefits. We also acknowledge the importance of careful consideration of the method used for performing an economic analysis. While the assumptions used in our study differ from others recently published [51, 52], we believe that the assumptions behind our estimates are solid and our bias is somewhat controlled as we cite official public pricing. Finally, we realize that the use of estimated incidence rates reported by Centers for Disease Control and Prevention (United States of America) for indirect standardisation of incidence rates for Mexico may not be ideal; however, we thought this was necessary because the SISVEFLU database likely underestimates the number of cases from primary care clinics, lacks the necessary population representation and most type B influenza cases reported in the database do not have lineage analysis (Additional file 1**: Supplement 1; Text S1.2**). We decided to use data from the US (estimated illness rate only) given the data quality and availability, and because health authorities in both countries recognise that infectious disease dynamics in the region, particularly those in which person-to-person transmission is paramount, exhibit similar epidemiological behaviour [53], possibly owing to the extent of human exchange between the two countries. Retrospective analyses have shown that in the case of the 2009 pandemic influenza, identification in the region could be traced back to near simultaneous origins in both the US and Mexico [54]. More importantly, the general epidemiological behaviour of infectious diseases in the last decade, in terms of timing and viral type, has been very similar [55]. As it is epidemiologically accepted to use surrogate data for indirect rate standardisation when local data are unavailable, provided there are reasonable grounds for doing so, we believe that, because the US is a neighbouring country in the same hemisphere and there are documented similarities in the epidemiological behaviour of the disease in both countries, our decision for considering data from the CDC as the best available source for estimating the incidence of influenza in Mexico is supported.

## Conclusions

Our study suggests that influenza in the school-aged population has a relatively low incidence, lethality, and mortality, likely due to a cohort effect resulting from at least a decade of vaccinating against influenza in Mexico. Although the true incidence is probably underestimated, surrogate data allowed us to estimate a reasonable burden of disease for the Mexican population in the last decade, ranging from 0.5 to 2.1 million cases each year for the school-aged population, which represents a substantial economic impact for the health care system. In addition, economic analysis showed that vaccination of the school-aged population is cost-saving. Because this age group represents influenza “superspreaders” and vaccination is shown to be a cost-effective intervention, expanding the current vaccination schedule to include this age group is supported. The national estimates for Mexico as a middle-income country provided from this study will be of great value for health care decision makers in middle-income countries.

## Supplementary information

**Additional file 1 Supplement 1 Text S1.1.** ICD-10 codes used for case selection. **Text S1.2.** Methodology for calculation of national estimates. **Figure S1.** Flowchart of key decision-making processes to validate the quality and relevance of data **Table S1.1.** Availability of datasets used in the study by year. **Table S1.2.** Influenza cases per 100,000 people in the United States by age group. **Table S1.3.** Estimation of influenza cases in Mexico. **Table S1.4.** Estimation of influenza cases in Mexico by state. **Table S1.5.** Estimation of influenza cases in Mexico by state and age group. **Supplement 2 Text S2.** Methodology for the estimation of different scenarios. **Table S2.1.** Confirmed cases of influenza per season and institution, all ages. **Table S2.2.** Incidence of influenza in the United States by age group. **Table S2.3.** Estimated cases of influenza in Mexico, per season. **Table S2.4.** Estimated cases of influenza in Mexico per season and age group. **Table S2.5.** Likelihood of seeking medical care by age group (scenario 0). **Table S2.6.** Classification of cases in each scenario according to health outcome. **Table S2.7.** Health outcomes and classification by scenario, results from SISVEFLU. **Table S2.8.** Estimated cases of influenza per scenario and season. **Table S2.9.** Likelihood of occurrence of each scenario for a typical season. **Supplement 3 Table S3.1.** Unit costs using 2018 prices from the three largest public health care providers in Mexico. **Table S3.2.** Unit cost by public health provider and season. **Table S3.3.** Affiliated population by public health provider and season. **Table S3.4.** Percentage of population by public health provider and per season. **Table S3.5.** Average weighted cost by institution. **Table S3.6.** National Consumer Price Index (INPC) at baseline year 2018. **Supplement 4**. **Figure S2.** Projection of school-aged population and total population in Mexico (2010–2030). **Table S4.** Effectiveness of influenza vaccination in the Northern Hemisphere. **Supplement 5 Table S5.1.** Confirmed influenza cases by age group and season. **Table S5.2.** Estimated influenza cases by age group and season. **Table S5.3.** Incidence per 100,000 inhabitants by age group and season. **Table S5.4.** Lethality by age group and season. **Table S5.5.** Years of life lost per age group by season. **Table S5.6.** Estimated influenza cases by age and season.

## Abbreviations

ER: Emergency room
ICD-10: International Classification of Diseases, 10th revision
IIOP: Intense influenza outbreak period
ILI: Influenza-like illness
MAARI: Medically attended acute respiratory illness
MXN: Mexican pesos
PCR: Polymerase chain reaction
QALY: Quality adjusted life year
RR: Relative risk
SAEH: Automated Hospital Discharge System
SAGE: Strategic Advisory Group of Experts
SARI: Severe acute respiratory illness
SEED: National Death Epidemiological and Statistics Subsystem
SISVEFLU: Mexico’s Influenza Surveillance System
USD: United States dollars
WHO: World Health Organization
YLL: Years of life lost
