# Supplementary material for: Influenza in the school-aged population in Mexico: burden of disease and cost-effectiveness of vaccination in children
Source: BMC Infect Dis. 2020 Mar 20;20:240. doi: 10.1186/s12879-020-4948-5 (PMC7085158; doi:10.1186/s12879-020-4948-5)
Supplement: Supplementary file 1 — Additional file 1 Supplement 1 Text S1.1. ICD-10 codes used for case selection. Text S1.2. Methodology for calculation of national estimates. Figure S1. Flowchart of key decision-making processes to validate the quality and relevance of data Table S1.1. Availability of datasets used in the study by year. Table S1.2. Influenza cases per 100,000 people in the United States by age group. Table S1.3. Estimation of influenza cases in Mexico. Table S1.4. Estimation of influenza cases in Mexico by state. Table S1.5. Estimation of influenza cases in Mexico by state and age group. Supplement 2 Text S2. Methodology for the estimation of different scenarios. Table S2.1. Confirmed cases of influenza per season and institution, all ages. Table S2.2. Incidence of influenza in the United States by age group. Table S2.3. Estimated cases of influenza in Mexico, per season. Table S2.4. Estimated cases of influenza in Mexico per season and age group. Table S2.5. Likelihood of seeking medical care by age group (scenario 0). Table S2.6. Classification of cases in each scenario according to health outcome. Table S2.7. Health outcomes and classification by scenario, results from SISVEFLU. Table S2.8. Estimated cases of influenza per scenario and season. Table S2.9. Likelihood of occurrence of each scenario for a typical season. Supplement 3 Table S3.1. Unit costs using 2018 prices from the three largest public health care providers in Mexico. Table S3.2. Unit cost by public health provider and season. Table S3.3. Affiliated population by public health provider and season. Table S3.4. Percentage of population by public health provider and per season. Table S3.5. Average weighted cost by institution. Table S3.6. National Consumer Price Index (INPC) at baseline year 2018. Supplement 4. Figure S2. Projection of school-aged population and total population in Mexico (2010–2030). Table S4. Effectiveness of influenza vaccination in the Northern Hemisphere. Supplement 5 Table S5.1. Confirmed influ [file 12879_2020_4948_MOESM1_ESM.docx]

# ADDITIONAL FILE 1

# Influenza in the school-aged population in Mexico: burden of disease and cost-effectiveness of vaccination in children

Jorge Abelardo Falcón-Lezama^1^, Rodrigo Saucedo-Martínez^1^, Miguel Betancourt-Cravioto^1^, Myrna María Alfaro-Cortes^1^, Roberto Isaac Bahena-González^1^, Roberto Tapia-Conyer^1,2*^

^1^Sociedad Mexicana de Salud Pública, Herschel 109, Anzures, Miguel Hidalgo, 11590 Mexico City, Mexico

^2^Universidad Nacional Autónoma de México, Av Universidad 3000, Circuito Escolar CU, Edificio B 1er Piso, Coyoacan, 04510 Mexico City, Mexico

**^*^Corresponding author:**

Roberto Tapia-Conyer

Lago Zurich 245, Presa Falcon Floor 20, Col. Ampliacion Granada, Miguel Hidalgo, 11529 Mexico City, Mexico

Tel: +52 (55) 5339 1796

Email: tapiaconyer@yahoo.com.mx

# Supplement 1

## Text S1.1. ICD-10 codes used for case selection

For influenza records from the databases of the National Mortality Epidemiological and Statistical System (SEED) and the Automated Hospital Discharge System (SAEH), the International Statistical Classification of Diseases and Related Health Problems, 10th revision, (ICD-10) codes that were used for the selection of cases were: J09x, influenza due to certain identified influenza virus; J100, influenza with pneumonia, other influenza virus identified; J101, influenza with other respiratory manifestations, other influenza virus identified; J108, influenza with other manifestations, other influenza virus identified; J110, influenza with pneumonia, virus not identified; J111 influenza with other respiratory manifestations, virus not identified; and J18, influenza with other manifestations, virus not identified.

## Text S1.2. Methodology for calculation of national estimates

**Main characteristics of SISVEFLU**

The SISVEFLU (Influenza Epidemiologic Surveillance System) was developed by the General Directorate of Epidemiology of Mexico’s Ministry of Health with the objective of collecting epidemiologic data about trends in circulating strains of influenza virus, as well as other causative agents of severe acute respiratory infection (SARI) [1].

The SISVEFLU system operates through monitoring health facilities that were selected for convenience. Because of this, the collected data are not considered to be representative of the population. Facilities, both primary health care clinics and hospitals, must comply with at least one of the following criteria [2].

- They must be willing to be part of the SISVEFLU.
- They serve a relatively large population.
- They have adequate medical staff sufficiently specialised to diagnose, clinically manage, and report influenza cases.
- They have, or have access to, a high-quality diagnostic laboratory.

**Burden of disease estimation**

SISVEFLU’s ultimate goal is to identify and confirm influenza cases and trends, as well as deaths, attributed to both influenza and other SARIs. According to the epidemiological surveillance guidelines, sample collection for confirmation is considered in only 10% of cases in primary health care facilities, whereas in secondary and tertiary health care facilities, samples must be submitted for confirmation in 100% of cases. In light of this, reporting of cases from primary care clinics may be underestimated compared with that reported from hospitals. Unlike primary care clinics, hospitals do not have a defined population because they receive cases from different geographical areas. Thus, information from these facilities cannot be extrapolated. According to the World Health Organisation’s methodology to estimate burden of disease due to influenza, data extrapolation from the SISVEFLU is limited because it is not representative of the population (**Figure S1**) [2]. Moreover, as noted from the data collected and reported in the article, most type B influenza cases did not have lineage analysis; therefore, data cannot be extrapolated for either the Victoria or Yamagata lineages.

As an alternative, we used the standardised influenza incidence data for the United States, estimated by the Centers for Disease Control and Prevention (CDC) for the same seasons (**Table S1.2**). We chose the United States because it is in the Northern Hemisphere and shares viral circulation with Mexico.

**Tables S1.3**, **S1.4,** and **S1.5** show national and state estimates obtained by indirectly standardising CDC estimates to the population of Mexico, taking the same age groups into consideration. For this exercise, data on Mexican population projections for the study period were obtained from the National Population Council database at the beginning of each year [3].

**References for Text S1**

1. Dirección General de Epidemiología. Manual para la Vigilancia Epidemiológica de Influenza [Manual for epidemiological influenza surveillance]. 2014. http://187.191.75.115/gobmx/salud/documentos/manuales/12_Manual_VE_Influenza.pdf. Accessed 24 September 2019. Spanish.
2. World Health Organization. Sentinel surveillance. [https://www.who.int/immunisation/monitoring_surveillance/burden/vpd/surveillance_type/sentinel/en/](https://www.who.int/immunization/monitoring_surveillance/burden/vpd/surveillance_type/sentinel/en/). Accessed 29 March 2019.
3. Consejo Nacional de Población–CONAPO. <https://datos.gob.mx/busca/dataset/proyecciones-de-la-poblacion-de-mexico-y-de-las-entidades-federativas-2016-2050>. Accessed 18 January 2019. Spanish.

## Figure S1. Flowchart of key decision-making processes to validate the quality and relevance of data


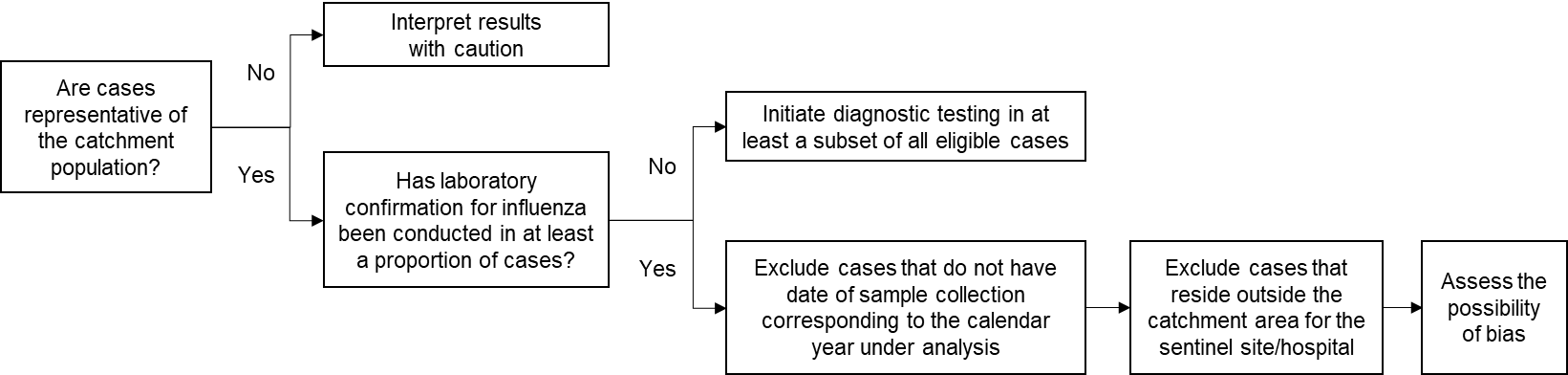


Source: Adapted from WHO (World Health Organization, 2015)

## Table S1.1. Availability of datasets used in the study by year

| **Database** | **2009** | **2010** | **2011** | **2012** | **2013** | **2014** | **2015** | **2016** | **2017** | **2018** | **Notes** |
| --- | --- | --- | --- | --- | --- | --- | --- | --- | --- | --- | --- |
| SISVEFLU |  |  |  |  |  |  |  |  |  |  | Surveillance system |
| SEED | N/A |  |  |  |  |  |  | N/A | N/A | N/A | Registry of deaths |
| SAEH | N/A |  |  |  |  |  |  |  | N/A | N/A | Registry of hospital discharges |

Abbreviations: N/A, not available; SAEH, Automated Hospital Discharge System; SEED, National Death Epidemiological and Statistics Subsystem; SISVEFLU: Mexico’s Influenza Surveillance System

## Table S1.2. Influenza cases per 100,000 people in the United States by age group

| **Season** | **0–4 years** | **5–17 years** | **18–49 years** | **50–64 years** | **≥65 years** |
| --- | --- | --- | --- | --- | --- |
| **2010–2011** | 13,743.20 | 8216.60 | 5468.10 | 8240.50 | 4521.10 |
| **2011–2012** | 4697.10 | 3711.80 | 2564.20 | 3181.40 | 2333.60 |
| **2012–2013** | 17,820.50 | 12,419.20 | 8383.60 | 12,852.10 | 9712.10 |
| **2013–2014** | 12,711.70 | 7416.10 | 9589.60 | 13,712.90 | 3819.30 |
| **2014–2015** | 16,135.90 | 11,895.00 | 6310.30 | 11,626.40 | 10,120.20 |
| **2015–2016** | 11,498.70 | 8094.10 | 7390.70 | 11,200.80 | 3460.60 |
| **2016–2017** | 11,478.30 | 11,083.30 | 6227.90 | 13,654.80 | 9013.80 |
| **2017–2018** | 19,983.70 | 13,985.60 | 10,469.70 | 24,588.10 | 14,371.40 |
| **2018–2019^a^** | 13,508.64 | 9602.71 | 7050.51 | 12,382.13 | 7169.01 |

^a^Incidence for season 2018–2019 was estimated for each age group as the average of seasons 2010–2011 to 2017–2018.

*Source:* Centers for Disease Control and Prevention. Disease Burden of Influenza. https://www.cdc.gov/flu/about/burden/index.html. Accessed 18 May 2019.

## Table S1.3. Estimation of influenza cases in Mexico

| **Season** | **Population** | **Estimated cases** | **Cases**  **per 100,000** |
| --- | --- | --- | --- |
| **2010–2011** | 114,551,762 | 8,275,119 | 7223.91 |
| **2011–2012** | 116,179,714 | 3,616,140 | 3112.54 |
| **2012–2013** | 117,668,241 | 12,803,672 | 10881.16 |
| **2013–2014** | 119,216,240 | 11,281,198 | 9462.80 |
| **2014–2015** | 120,653,293 | 11,413,739 | 9459.95 |
| **2015–2016** | 122,038,924 | 9,928,898 | 8135.85 |
| **2016–2017** | 123,388,022 | 11,074,556 | 8975.39 |
| **2017–2018** | 124,692,044 | 17,749,078 | 14,234.33 |
| **2018–2019** | 125,960,168 | 11,223,972 | 8910.73 |
| **TOTAL** | | **97,366,372** |  |

## Table S1.4. Estimation of influenza cases in Mexico by state

| **State** | **2010 –2011** | **2011–2012** | **2012–2013** | **2013–2014** | **2014–2015** | **2015–2016** | **2016–2017** | **2017–2018** | **2018–2019** | **Total** |
| --- | --- | --- | --- | --- | --- | --- | --- | --- | --- | --- |
| **Aguascalientes** | 88,468 | 38,893 | 138,436 | 122,290 | 125,380 | 109,946 | 122,417 | 195,709 | 125,224 | **1,066,763** |
| **Baja California** | 230,535 | 100,611 | 353,751 | 315,400 | 312,199 | 275,482 | 305,179 | 493,168 | 315,036 | **2,701,361** |
| **Baja California Sur** | 46,169 | 20,463 | 73,047 | 66,271 | 66,337 | 59,475 | 66,012 | 107,321 | 69,156 | **574,250** |
| **Campeche** | 60,693 | 26,725 | 95,081 | 84,619 | 85,695 | 75,387 | 83,980 | 135,458 | 86,767 | **734,406** |
| **Chiapas** | 365,348 | 159,527 | 566,391 | 488,729 | 513,013 | 440,813 | 492,995 | 782,583 | 506,517 | **4,315,916** |
| **Chihuahua** | 252,216 | 109,645 | 386,237 | 339,291 | 341,191 | 296,272 | 329,974 | 528,555 | 334,465 | **2,917,846** |
| **Coahuila** | 203,331 | 88,990 | 315,962 | 279,440 | 283,165 | 247,187 | 276,269 | 444,876 | 283,098 | **2,422,318** |
| **Colima** | 47,408 | 20,906 | 74,566 | 66,673 | 67,266 | 59,306 | 66,213 | 107,070 | 68,230 | **577,638** |
| **Ciudad de México** | 626,024 | 272,413 | 958,372 | 860,627 | 830,047 | 725,357 | 805,871 | 1,302,691 | 793,820 | **7,175,222** |
| **Durango** | 121,719 | 53,215 | 188,964 | 164,776 | 169,746 | 146,398 | 163,687 | 260,589 | 165,172 | **1,434,266** |
| **Guerrero** | 254,561 | 110,243 | 388,873 | 331,818 | 345,836 | 292,568 | 328,247 | 517,711 | 326,742 | **2,896,598** |
| **Guanajuato** | 407,406 | 177,793 | 628,644 | 549,226 | 560,887 | 486,267 | 540,340 | 861,232 | 548,143 | **4,759,938** |
| **Hidalgo** | 196,532 | 85,981 | 305,480 | 268,000 | 274,224 | 237,469 | 267,063 | 428,214 | 271,118 | **2,334,079** |
| **Jalisco** | 544,764 | 237,777 | 841,824 | 739,591 | 750,109 | 651,298 | 726,082 | 1,162,135 | 737,133 | **6,390,713** |
| **México** | 1,116,748 | 488,023 | 1,723,417 | 1,536,580 | 1,530,724 | 1,349,312 | 1,495,462 | 2,407,674 | 1,523,682 | **13,171,621** |
| **Michoacán** | 321,865 | 140,325 | 497,058 | 430,590 | 443,671 | 379,657 | 425,372 | 676,765 | 427,974 | **3,743,277** |
| **Morelos** | 129,869 | 56,918 | 202,305 | 178,719 | 181,070 | 157,340 | 176,832 | 284,736 | 179,177 | **1,546,967** |
| **Nayarit** | 80,088 | 35,145 | 125,448 | 110,111 | 113,351 | 98,143 | 110,434 | 177,291 | 112,856 | **962,868** |
| **Nuevo León** | 339,695 | 149,960 | 534,871 | 481,147 | 482,418 | 427,511 | 475,687 | 768,696 | 487,869 | **4,147,854** |
| **Oaxaca** | 282,272 | 122,630 | 433,494 | 372,058 | 385,496 | 326,933 | 368,345 | 584,162 | 367,734 | **3,243,123** |
| **Puebla** | 431,890 | 188,404 | 666,817 | 580,015 | 596,147 | 513,832 | 574,243 | 914,328 | 581,259 | **5,046,934** |
| **Querétaro** | 134,677 | 59,520 | 212,113 | 189,718 | 192,312 | 170,629 | 189,587 | 305,903 | 196,741 | **1,651,201** |
| **Quintana Roo** | 97,362 | 43,195 | 153,662 | 139,994 | 139,453 | 126,315 | 138,737 | 225,379 | 146,883 | **1,210,978** |
| **San Luis Potosí** | 191,638 | 83,404 | 294,723 | 255,402 | 261,996 | 224,382 | 252,060 | 401,475 | 253,032 | **2,218,110** |
| **Sinaloa** | 203,401 | 89,134 | 316,571 | 279,494 | 283,456 | 246,487 | 275,743 | 441,583 | 277,721 | **2,413,591** |
| **Sonora** | 195,348 | 85,477 | 303,190 | 268,652 | 270,846 | 236,800 | 264,370 | 425,038 | 269,000 | **2,318,724** |
| **Tabasco** | 164,888 | 72,148 | 255,246 | 225,650 | 228,068 | 199,645 | 221,144 | 354,221 | 225,715 | **1,946,724** |
| **Tamaulipas** | 239,711 | 104,631 | 370,115 | 326,898 | 329,037 | 286,170 | 319,070 | 511,736 | 322,513 | **2,809,881** |
| **Tlaxcala** | 86,924 | 38,129 | 135,331 | 119,242 | 121,696 | 106,327 | 118,452 | 189,594 | 120,799 | **1,036,492** |
| **Veracruz** | 560,390 | 245,095 | 870,594 | 765,261 | 777,728 | 672,094 | 754,565 | 1,208,796 | 756,174 | **6,610,696** |
| **Yucatán** | 142,469 | 62,557 | 221,918 | 196,827 | 197,971 | 173,096 | 193,080 | 310,474 | 196,259 | **1,694,652** |
| **Zacatecas** | 110,709 | 48,263 | 171,171 | 148,088 | 153,205 | 131,002 | 147,044 | 233,914 | 147,965 | **1,291,361** |
| **TOTAL** | **8,275,119** | **3,616,140** | **12,803,672** | **11,281,198** | **11,413,739** | **9,928,898** | **11,074,556** | **17,749,078** | **11,223,972** | **97,366,372** |

## Table S1.5. Estimation of influenza cases in Mexico by state and age group

| **State** | **Age group, years** | **2010–2011** | **2011–2012** | **2012–2013** | **2013–2014** | **2014–2015** | **2015–2016** | **2016–2017** | **2017–2018** | **2018–2019** | **Total** |
| --- | --- | --- | --- | --- | --- | --- | --- | --- | --- | --- | --- |
| **Aguascalientes** | 0–4 | 17,541 | 6011 | 22,841 | 16,325 | 20,754 | 14,805 | 14,660 | 25,338 | 16,980 | **155,255** |
|  | 5–17 | 26,896 | 12,282 | 41,499 | 25,025 | 40,520 | 27,834 | 38,159 | 48,184 | 33,084 | **293,482** |
|  | 18–49 | 31,216 | 15,043 | 50,441 | 59,140 | 39,819 | 47,668 | 40,898 | 69,931 | 47,858 | **402,015** |
|  | 50–64 | 10,010 | 4053 | 17,143 | 19,136 | 16,949 | 17,034 | 21,620 | 40,464 | 21,152 | **167,560** |
|  | ≥65 | 2804 | 1505 | 6512 | 2664 | 7338 | 2605 | 7080 | 11,792 | 6151 | **48,451** |
| **Baja California** | 0–4 | 42,531 | 14,424 | 54,245 | 38,368 | 48,272 | 34,078 | 34,150 | 59,588 | 40,284 | **365,941** |
|  | 5–17 | 65,855 | 29,684 | 99,017 | 58,954 | 94,258 | 63,940 | 87,747 | 110,965 | 76,340 | **686,759** |
|  | 18–49 | 88,389 | 41,941 | 138,485 | 159,920 | 106,069 | 125,096 | 107,213 | 183,115 | 125,174 | **1,075,402** |
|  | 50–64 | 27,075 | 10,959 | 46,346 | 51,725 | 45,802 | 46,022 | 58,685 | 110,329 | 57,911 | **454,854** |
|  | ≥65 | 6684 | 3603 | 15,658 | 6433 | 17,799 | 6346 | 17,384 | 29,171 | 15,327 | **118,406** |
| **Baja California Sur** | 0–4 | 8564 | 2973 | 11,441 | 8280 | 10,654 | 7692 | 7793 | 13,720 | 9364 | **80,481** |
|  | 5–17 | 12,882 | 5905 | 20,030 | 12,124 | 19,701 | 13,581 | 18,795 | 23,973 | 16,635 | **143,625** |
|  | 18–49 | 17,972 | 8651 | 28,979 | 33,946 | 22,838 | 27,320 | 23,543 | 40,423 | 27,770 | **231,442** |
|  | 50–64 | 5454 | 2221 | 9449 | 10,606 | 9445 | 9543 | 12,208 | 23,038 | 12,146 | **94,108** |
|  | ≥65 | 1297 | 712 | 3148 | 1315 | 3699 | 1340 | 3673 | 6167 | 3241 | **24,593** |
| **Campeche** | 0–4 | 11,306 | 3926 | 15,111 | 10,938 | 14,078 | 10,165 | 10,231 | 17,894 | 12,127 | **105,775** |
|  | 5–17 | 17,576 | 7980 | 26,809 | 16,077 | 25,888 | 17,686 | 24,384 | 31,010 | 21,475 | **188,886** |
|  | 18–49 | 22,356 | 10,730 | 35,832 | 41,848 | 28,070 | 33,480 | 28,763 | 49,244 | 33,741 | **284,064** |
|  | 50–64 | 7316 | 2944 | 12,375 | 13,732 | 12,091 | 12,082 | 15,273 | 28,491 | 14,853 | **119,157** |
|  | ≥65 | 2139 | 1146 | 4954 | 2024 | 5568 | 1974 | 5329 | 8818 | 4571 | **36,523** |
| **Chiapas** | 0–4 | 80,023 | 27,829 | 107,285 | 77,767 | 100,237 | 72,475 | 72,592 | 126,525 | 85,557 | **750,289** |
|  | 5–17 | 117,934 | 53,702 | 180,961 | 108,831 | 175,747 | 120,402 | 166,251 | 211,609 | 146,576 | **1,282,014** |
|  | 18–49 | 120,389 | 57,744 | 192,716 | 224,910 | 150,742 | 179,650 | 154,039 | 263,272 | 180,116 | **1,523,578** |
|  | 50–64 | 36,276 | 14,534 | 60,847 | 67,232 | 58,942 | 58,642 | 74,133 | 138,290 | 72,091 | **580,987** |
|  | ≥65 | 10,726 | 5718 | 24,581 | 9990 | 27,344 | 9645 | 25,979 | 42,888 | 22,177 | **179,048** |
| **Chihuahua** | 0–4 | 47,948 | 16,217 | 60,821 | 42,901 | 53,822 | 37,886 | 37,510 | 64,778 | 43,412 | **405,294** |
|  | 5–17 | 72,808 | 32,866 | 109,794 | 65,467 | 104,826 | 71,213 | 97,401 | 122,668 | 83,978 | **761,021** |
|  | 18–49 | 91,301 | 43,275 | 142,726 | 164,618 | 109,046 | 128,439 | 109,334 | 185,539 | 126,059 | **1,100,336** |
|  | 50–64 | 31,101 | 12,489 | 52,401 | 58,030 | 50,990 | 50,845 | 64,321 | 119,986 | 62,501 | **502,665** |
|  | ≥65 | 9058 | 4798 | 20,495 | 8276 | 22,507 | 7888 | 21,408 | 35,584 | 18,516 | **148,529** |
| **Coahuila** | 0–4 | 38,611 | 13,302 | 50,818 | 36,513 | 46,660 | 33,455 | 33,862 | 59,583 | 40,650 | **353,453** |
|  | 5–17 | 58,133 | 26,408 | 88,775 | 53,266 | 85,821 | 58,664 | 80,544 | 101,953 | 70,258 | **623,823** |
|  | 18–49 | 73,515 | 35,031 | 116,158 | 134,702 | 89,716 | 106,252 | 90,608 | 154,032 | 104,825 | **904,839** |
|  | 50–64 | 25,754 | 10,348 | 43,451 | 48,150 | 42,337 | 42,245 | 53,428 | 99,671 | 51,940 | **417,325** |
|  | ≥65 | 7319 | 3900 | 16,761 | 6809 | 18,630 | 6569 | 17,827 | 29,636 | 15,426 | **122,878** |
| **Colima** | 0–4 | 8541 | 2963 | 11,397 | 8243 | 10,601 | 7649 | 7704 | 13,489 | 9150 | **79,736** |
|  | 5–17 | 13,224 | 6027 | 20,328 | 12,236 | 19,777 | 13,561 | 18,697 | 23,766 | 16,442 | **144,058** |
|  | 18–49 | 17,615 | 8452 | 28,215 | 32,941 | 22,088 | 26,335 | 22,565 | 38,531 | 26,333 | **223,075** |
|  | 50–64 | 6188 | 2485 | 10,429 | 11,551 | 10,151 | 10,124 | 12,815 | 23,927 | 12,479 | **100,151** |
|  | ≥65 | 1839 | 979 | 4198 | 1702 | 4649 | 1636 | 4432 | 7356 | 3826 | **30,617** |
| **Ciudad de México** | 0–4 | 89,835 | 29,880 | 110,130 | 76,297 | 93,949 | 64,864 | 63,263 | 107,729 | 71,427 | **707,373** |
|  | 5–17 | 150,619 | 66,732 | 218,704 | 127,892 | 200,753 | 133,645 | 179,367 | 221,713 | 148,990 | **1,448,415** |
|  | 18–49 | 249,286 | 117,099 | 382,693 | 437,326 | 286,986 | 334,813 | 281,170 | 470,667 | 315,385 | **2,875,424** |
|  | 50–64 | 103,760 | 41,293 | 171,708 | 188,456 | 164,122 | 162,208 | 201,867 | 370,522 | 189,967 | **1,593,902** |
|  | ≥65 | 32,524 | 17,409 | 75,138 | 30,656 | 84,237 | 29,827 | 80,204 | 132,061 | 68,052 | **550,108** |
| **Durango** | 0–4 | 23,799 | 8203 | 31,354 | 22,539 | 28,816 | 20,671 | 20,292 | 34,699 | 22,989 | **213,364** |
|  | 5–17 | 36,470 | 16,550 | 55,577 | 33,312 | 53,615 | 36,611 | 50,231 | 63,512 | 43,686 | **389,562** |
|  | 18–49 | 41,973 | 20,047 | 66,624 | 77,432 | 51,684 | 61,344 | 52,269 | 88,782 | 60,372 | **520,528** |
|  | 50–64 | 14,674 | 5,874 | 24,569 | 27,123 | 23,758 | 23,617 | 29,745 | 55,263 | 28,684 | **233,305** |
|  | ≥65 | 4803 | 2541 | 10,841 | 4371 | 11,873 | 4155 | 11,150 | 18,333 | 9441 | **77,508** |
| **Guerrero** | 0–4 | 52,006 | 17,711 | 66,883 | 47,508 | 60,026 | 42,557 | 41,822 | 71,668 | 47,578 | **447,758** |
|  | 5–17 | 80,876 | 36,289 | 120,495 | 71,410 | 113,639 | 76,720 | 104,600 | 131,398 | 89,781 | **825,210** |
|  | 18–49 | 82,308 | 39,165 | 129,674 | 150,140 | 99,836 | 118,044 | 100,312 | 169,929 | 115,244 | **1,004,653** |
|  | 50–64 | 29,004 | 11,586 | 48,366 | 53,290 | 46,591 | 46,228 | 57,576 | 105,778 | 54,297 | **452,716** |
|  | ≥65 | 10,367 | 5491 | 23,454 | 9469 | 25,744 | 9019 | 23,937 | 38,938 | 19,842 | **166,261** |
| **Guanajuato** | 0–4 | 80,423 | 27,454 | 103,932 | 74,005 | 93,732 | 66,615 | 66,350 | 115,061 | 77,279 | **704,852** |
|  | 5–17 | 123,571 | 55,772 | 186,280 | 111,055 | 177,791 | 120,761 | 164,990 | 207,828 | 142,485 | **1,290,533** |
|  | 18–49 | 142,216 | 68,116 | 227,020 | 264,601 | 177,123 | 210,831 | 179,675 | 305,127 | 207,386 | **1,782,095** |
|  | 50–64 | 46,220 | 18,543 | 77,737 | 86,012 | 75,512 | 75,234 | 94,894 | 176,575 | 91,804 | **742,531** |
|  | ≥65 | 14,976 | 7909 | 33,674 | 13,552 | 36,730 | 12,826 | 34,430 | 56,641 | 29,188 | **239,927** |
| **Hidalgo** | 0–4 | 37,250 | 12,721 | 48,171 | 34,312 | 43,473 | 30,907 | 30,805 | 53,483 | 36,028 | **327,150** |
|  | 5–17 | 57,599 | 26,098 | 87,512 | 52,377 | 84,180 | 57,401 | 78,889 | 99,872 | 68,751 | **612,679** |
|  | 18–49 | 68,884 | 32,909 | 109,403 | 127,194 | 84,935 | 100,853 | 86,068 | 146,416 | 99,718 | **856,380** |
|  | 50–64 | 24,832 | 9,991 | 42,010 | 46,620 | 41,051 | 41,024 | 51,730 | 96,217 | 49,998 | **403,473** |
|  | ≥65 | 7967 | 4262 | 18,384 | 7496 | 20,585 | 7285 | 19,571 | 32,225 | 16,622 | **134,397** |
| **Jalisco** | 0–4 | 104,704 | 35,646 | 134,570 | 95,557 | 120,694 | 85,540 | 85,031 | 147,261 | 98,719 | **907,722** |
|  | 5–17 | 157,299 | 71,241 | 238,773 | 142,842 | 229,468 | 156,400 | 214,139 | 270,162 | 185,408 | **1,665,733** |
|  | 18–49 | 194,380 | 92,817 | 308,404 | 358,374 | 239,177 | 283,847 | 242,188 | 411,864 | 280,365 | **2,411,414** |
|  | 50–64 | 67,224 | 26,846 | 112,027 | 123,381 | 107,817 | 106,921 | 134,657 | 250,255 | 129,981 | **1,059,108** |
|  | ≥65 | 21,156 | 11,228 | 48,051 | 19,437 | 52,954 | 18,590 | 50,067 | 82,593 | 42,660 | **346,736** |
| **México** | 0–4 | 205,667 | 69,545 | 260,760 | 183,885 | 230,636 | 162,308 | 161,763 | 280,741 | 188,967 | **1,744,272** |
|  | 5–17 | 319,827 | 144,061 | 480,203 | 285,707 | 456,473 | 309,432 | 422,744 | 532,109 | 364,291 | **3,314,848** |
|  | 18–49 | 417,401 | 199,068 | 660,678 | 766,863 | 511,253 | 606,110 | 516,270 | 876,460 | 595,553 | **5,149,656** |
|  | 50–64 | 138,268 | 56,040 | 237,310 | 265,205 | 235,148 | 236,594 | 299,744 | 559,985 | 292,146 | **2,320,441** |
|  | ≥65 | 35,585 | 19,309 | 84,465 | 34,920 | 97,213 | 34,867 | 94,941 | 158,379 | 82,725 | **642,404** |
| **Michoacán** | 0–4 | 62,146 | 21,415 | 81,829 | 58,805 | 75,162 | 53,903 | 53,507 | 92,403 | 61,920 | **561,090** |
|  | 5–17 | 96,100 | 43,358 | 144,770 | 86,278 | 138,075 | 93,750 | 128,422 | 162,219 | 111,538 | **1,004,510** |
|  | 18–49 | 110,256 | 52,491 | 173,890 | 201,455 | 134,044 | 158,597 | 134,730 | 228,114 | 154,611 | **1,348,188** |
|  | 50–64 | 39,235 | 15,624 | 65,011 | 71,396 | 62,211 | 61,515 | 77,030 | 142,294 | 73,444 | **607,760** |
|  | ≥65 | 14,128 | 7,436 | 31,558 | 12,656 | 34,179 | 11,891 | 31,685 | 51,735 | 26,461 | **221,730** |
| **Morelos** | 0–4 | 22,968 | 7,858 | 29,811 | 21,272 | 27,001 | 19,231 | 19,310 | 33,745 | 22,871 | **204,066** |
|  | 5–17 | 36,749 | 16,576 | 55,332 | 32,968 | 52,747 | 35,807 | 48,959 | 61,716 | 42,345 | **383,200** |
|  | 18–49 | 46,771 | 22,328 | 74,175 | 86,173 | 57,499 | 68,220 | 58,141 | 98,758 | 67,145 | **579,211** |
|  | 50–64 | 17,653 | 7,084 | 29,704 | 32,876 | 28,873 | 28,779 | 36,183 | 67,085 | 34,737 | **282,974** |
|  | ≥65 | 5727 | 3072 | 13,284 | 5430 | 14,950 | 5303 | 14,239 | 23,432 | 12,078 | **97,516** |
| **Nayarit** | 0–4 | 15,502 | 5365 | 20,584 | 14,853 | 19,060 | 13,722 | 13,670 | 23,688 | 15,875 | **142,318** |
|  | 5–17 | 22,976 | 10,490 | 35,438 | 21,367 | 34,591 | 23,756 | 32,836 | 41,839 | 29,007 | **252,301** |
|  | 18–49 | 27,908 | 13,377 | 44,615 | 52,038 | 34,860 | 41,523 | 35,569 | 60,726 | 41,501 | **352,116** |
|  | 50–64 | 10,241 | 4084 | 17,017 | 18,714 | 16,329 | 16,168 | 20,392 | 37,956 | 19,746 | **160,648** |
|  | ≥65 | 3462 | 1829 | 7793 | 3138 | 8512 | 2974 | 7968 | 13,082 | 6728 | **55,486** |
| **Nuevo León** | 0–4 | 60,475 | 20,839 | 79,621 | 57,217 | 73,129 | 52,443 | 52,394 | 91,184 | 61,565 | **548,865** |
|  | 5–17 | 92,390 | 42,279 | 143,152 | 86,503 | 140,342 | 96,590 | 132,664 | 167,859 | 115,513 | **1,017,294** |
|  | 18–49 | 129,350 | 61,972 | 206,609 | 240,899 | 161,319 | 192,088 | 164,157 | 279,641 | 190,691 | **1,626,727** |
|  | 50–64 | 44,660 | 18,017 | 75,944 | 84,488 | 74,575 | 74,697 | 94,755 | 177,309 | 92,679 | **737,124** |
|  | ≥65 | 12,818 | 6853 | 29,545 | 12,041 | 33,053 | 11,692 | 31,716 | 52,703 | 27,421 | **217,844** |
| **Oaxaca** | 0–4 | 54,418 | 18,535 | 70,006 | 49,734 | 62,848 | 44,565 | 43,912 | 75,465 | 50,392 | **469,875** |
|  | 5–17 | 86,536 | 38,857 | 129,120 | 76,581 | 121,963 | 82,405 | 112,612 | 141,753 | 97,021 | **886,848** |
|  | 18–49 | 93,365 | 44,458 | 147,312 | 170,699 | 113,602 | 134,438 | 114,260 | 193,590 | 131,320 | **1,143,046** |
|  | 50–64 | 34,845 | 13,861 | 57,612 | 63,200 | 55,008 | 54,331 | 67,869 | 125,087 | 64,423 | **536,236** |
|  | ≥65 | 13,108 | 6919 | 29,443 | 11,843 | 32,075 | 11,193 | 29,692 | 48,267 | 24,577 | **207,118** |
| **Puebla** | 0–4 | 85,324 | 29,125 | 110,246 | 78,494 | 99,408 | 70,643 | 70,109 | 121,269 | 81,378 | **745,997** |
|  | 5–17 | 131,568 | 59,461 | 198,870 | 118,721 | 190,319 | 129,445 | 177,086 | 223,187 | 152,993 | **1,381,650** |
|  | 18–49 | 148,573 | 71,051 | 236,436 | 275,149 | 183,897 | 218,551 | 186,570 | 317,463 | 216,238 | **1,853,929** |
|  | 50–64 | 49,887 | 19,985 | 83,658 | 92,430 | 81,032 | 80,619 | 101,462 | 188,395 | 97,745 | **795,213** |
|  | ≥65 | 16,538 | 8782 | 37,606 | 15,221 | 41,492 | 14,574 | 39,014 | 64,013 | 32,904 | **270,146** |
| **Querétaro** | 0–4 | 25,556 | 8,822 | 33,763 | 24,302 | 31,112 | 22,347 | 22,441 | 39,228 | 26,587 | **234,158** |
|  | 5–17 | 39,886 | 18,183 | 61,334 | 36,926 | 59,694 | 40,940 | 56,325 | 71,424 | 49,282 | **433,993** |
|  | 18–49 | 49,714 | 24,026 | 80,785 | 94,980 | 64,126 | 76,975 | 66,276 | 113,681 | 78,018 | **648,580** |
|  | 50–64 | 15,235 | 6190 | 26,279 | 29,439 | 26,165 | 26,386 | 33,676 | 63,385 | 33,324 | **260,079** |
|  | ≥65 | 4286 | 2300 | 9953 | 4071 | 11,215 | 3981 | 10,870 | 18,185 | 9529 | **74,390** |
| **Quintana Roo** | 0–4 | 19,159 | 6612 | 25,299 | 18,206 | 23,302 | 16,733 | 16,977 | 29,939 | 20,419 | **176,645** |
|  | 5–17 | 28,040 | 12,858 | 43,620 | 26,409 | 42,925 | 29,596 | 40,950 | 52,220 | 36,230 | **312,847** |
|  | 18–49 | 38,715 | 18,719 | 62,968 | 74,072 | 50,038 | 60,100 | 51,960 | 89,490 | 61,665 | **507,726** |
|  | 50–64 | 9511 | 3928 | 16,939 | 19,261 | 17,364 | 17,752 | 22,947 | 43,726 | 23,258 | **174,686** |
|  | ≥65 | 1937 | 1079 | 4836 | 2046 | 5825 | 2133 | 5903 | 10,005 | 5310 | **39,074** |
| **San Luis Potosí** | 0–4 | 36,447 | 12,324 | 46,207 | 32,584 | 40,867 | 28,758 | 28,630 | 49,741 | 33,549 | **309,107** |
|  | 5–17 | 57,687 | 25,954 | 86,411 | 51,351 | 81,945 | 55,481 | 75,487 | 94,603 | 64,479 | **593,398** |
|  | 18–49 | 65,605 | 31,264 | 103,672 | 120,224 | 80,072 | 94,831 | 80,717 | 136,952 | 93,022 | **806,359** |
|  | 50–64 | 23,546 | 9428 | 39,446 | 43,560 | 38,169 | 37,956 | 47,645 | 88,233 | 45,653 | **373,635** |
|  | ≥65 | 8353 | 4435 | 18,987 | 7684 | 20,943 | 7355 | 19,580 | 31,946 | 16,329 | **135,611** |
| **Sinaloa** | 0–4 | 36,389 | 12,446 | 47,204 | 33,675 | 42,732 | 30,427 | 30,088 | 51,892 | 34,749 | **319,602** |
|  | 5–17 | 58,222 | 26,383 | 88,472 | 52,955 | 85,115 | 58,044 | 79,155 | 99,429 | 67,924 | **615,698** |
|  | 18–49 | 73,229 | 34,926 | 115,915 | 134,539 | 89,685 | 106,306 | 90,460 | 153,449 | 104,201 | **902,711** |
|  | 50–64 | 27,172 | 10,891 | 45,616 | 50,428 | 44,233 | 44,033 | 55,379 | 102,750 | 53,261 | **433,763** |
|  | ≥65 | 8390 | 4489 | 19,364 | 7897 | 21,691 | 7678 | 20,662 | 34,063 | 17,584 | **141,817** |
| **Sonora** | 0–4 | 35,921 | 12,261 | 46,408 | 33,040 | 41,841 | 29,732 | 29,500 | 51,046 | 34,253 | **314,003** |
|  | 5–17 | 55,529 | 25,190 | 84,563 | 50,669 | 81,525 | 55,653 | 76,259 | 96,257 | 66,076 | **591,720** |
|  | 18–49 | 70,934 | 33,834 | 112,295 | 130,341 | 86,887 | 102,990 | 88,002 | 149,903 | 102,220 | **877,406** |
|  | 50–64 | 25,641 | 10,293 | 43,176 | 47,802 | 41,996 | 41,871 | 52,795 | 98,166 | 50,982 | **412,722** |
|  | ≥65 | 7323 | 3900 | 16,749 | 6800 | 18,597 | 6553 | 17,814 | 29,667 | 15,470 | **122,873** |
| **Tabasco** | 0–4 | 31,701 | 10,912 | 41,647 | 29,897 | 38,172 | 27,346 | 27,188 | 47,007 | 31,540 | **285,410** |
|  | 5–17 | 48,664 | 21,984 | 73,495 | 43,857 | 70,275 | 47,776 | 65,624 | 83,103 | 57,274 | **512,053** |
|  | 18–49 | 60,179 | 28,728 | 95,434 | 110,877 | 73,988 | 87,797 | 74,798 | 127,020 | 86,341 | **745,163** |
|  | 50–64 | 19,040 | 7,678 | 32,353 | 35,981 | 31,751 | 31,796 | 40,198 | 74,980 | 39,077 | **312,855** |
|  | ≥65 | 5303 | 2846 | 12,315 | 5038 | 13,881 | 4929 | 13,335 | 22,112 | 11,484 | **91,243** |
| **Tamaulipas** | 0–4 | 44,470 | 15,194 | 57,566 | 41,024 | 52,002 | 36,989 | 36,688 | 63,405 | 42,563 | **389,900** |
|  | 5–17 | 66,805 | 30,271 | 101,506 | 60,754 | 97,645 | 66,583 | 91,201 | 115,051 | 78,916 | **708,730** |
|  | 18–49 | 88,599 | 41,932 | 138,095 | 159,049 | 105,208 | 123,744 | 104,901 | 177,309 | 119,999 | **1,058,836** |
|  | 50–64 | 30,650 | 12,327 | 51,808 | 57,464 | 50,574 | 50,511 | 63,815 | 118,899 | 61,869 | **497,918** |
|  | ≥65 | 9188 | 4908 | 21,141 | 8608 | 23,608 | 8343 | 22,465 | 37,072 | 19,165 | **154,497** |
| **Tlaxcala** | 0–4 | 16,914 | 5,771 | 21,838 | 15,542 | 19,675 | 13,977 | 13,957 | 24,280 | 16,384 | **148,339** |
|  | 5–17 | 26,035 | 11,813 | 39,664 | 23,772 | 38,258 | 26,122 | 35,748 | 45,074 | 30,915 | **277,401** |
|  | 18–49 | 30,964 | 14,868 | 49,681 | 58,052 | 38,957 | 46,487 | 39,714 | 67,621 | 46,087 | **392,430** |
|  | 50–64 | 9864 | 4000 | 16,949 | 18,952 | 16,812 | 16,924 | 21,468 | 40,159 | 20,978 | **166,106** |
|  | ≥65 | 3146 | 1676 | 7199 | 2923 | 7993 | 2817 | 7565 | 12,461 | 6435 | **52,216** |
| **Veracruz** | 0–4 | 98,986 | 33,920 | 128,894 | 92,125 | 117,119 | 83,548 | 83,076 | 143,752 | 96,453 | **877,872** |
|  | 5–17 | 159,385 | 71,841 | 239,638 | 142,678 | 228,115 | 154,738 | 211,121 | 265,519 | 181,734 | **1,654,771** |
|  | 18–49 | 198,653 | 94,573 | 313,301 | 362,974 | 241,526 | 285,780 | 242,265 | 409,385 | 276,931 | **2,425,388** |
|  | 50–64 | 77,807 | 31,135 | 130,189 | 143,683 | 125,828 | 125,053 | 156,631 | 289,351 | 149,301 | **1,228,977** |
|  | ≥65 | 25,559 | 13,625 | 58,571 | 23,801 | 65,140 | 22,974 | 61,473 | 100,790 | 51,756 | **423,688** |
| **Yucatán** | 0–4 | 24,754 | 8449 | 31,978 | 22,765 | 28,828 | 20,485 | 20,434 | 35,527 | 23,983 | **217,202** |
|  | 5–17 | 40,186 | 18,174 | 60,821 | 36,333 | 58,284 | 39,669 | 54,092 | 67,979 | 46,485 | **422,024** |
|  | 18–49 | 52,498 | 25,134 | 83,734 | 97,559 | 65,282 | 77,681 | 66,393 | 113,094 | 77,100 | **658,474** |
|  | 50–64 | 18,847 | 7519 | 31,348 | 34,492 | 30,112 | 29,833 | 37,574 | 69,858 | 36,309 | **295,893** |
|  | ≥65 | 6184 | 3281 | 14,038 | 5678 | 15,465 | 5428 | 14,588 | 24,017 | 12,381 | **101,060** |
| **Zacatecas** | 0–4 | 22,043 | 7563 | 28,774 | 20,590 | 26,208 | 18,718 | 18,562 | 32,044 | 21,449 | **195,951** |
|  | 5–17 | 32,950 | 14,910 | 49,928 | 29,843 | 47,899 | 32,617 | 44,753 | 56,604 | 38,953 | **348,456** |
|  | 18–49 | 37,644 | 17,936 | 59,467 | 68,953 | 45,919 | 54,377 | 46,165 | 78,126 | 52,934 | **461,522** |
|  | 50–64 | 13,146 | 5262 | 22,008 | 24,295 | 21,281 | 21,153 | 26,566 | 49,229 | 25,492 | **208,432** |
|  | ≥65 | 4926 | 2592 | 10,995 | 4407 | 11,898 | 4137 | 10,997 | 17,911 | 9138 | **77,000** |
| **TOTAL** | | **8,275,119** | **3,616,140** | **12,803,672** | **11,281,198** | **11,413,739** | **9,928,898** | **11,074,556** | **17,749,078** | **11,223,972** | **97,366,372** |

# Supplement 2

## Text S2. Methodology for the estimation of different scenarios

From seasons 2009–2010 to 2017–2018, health monitoring units registered 50,467 confirmed cases of influenza. These cases were distributed between the different institutions of Mexico’s Health System as shown in **Table S2.1**.

Given that SISVEFLU cases were recorded from monitoring facilities and the data are not defined as sentinel, data cannot be extrapolated directly to population-wide estimates. Therefore, the total number of influenza cases in Mexico were estimated by indirectly standardising reported values of influenza incidence in the United States for each season and age group (**Table S2.2**) into the Mexican population structure according to official population projections.

**Table S2.3** shows estimated cases of influenza in Mexico per season, considering both official population projections and influenza incidence from the United States.

**Table S2.4** shows estimated cases of influenza per age group. Disaggregated data from each age group allows us to capture differences in health outcomes more accurately; it also enables us to estimate herd effect, especially in those who are not vaccinated under the current schedule.

Estimated cases were then allocated into eight different scenarios, considering 1) the probability of not demanding medical care (scenario 0), as reported by Molinari et al. [1] for different age groups, 2) where cases were diagnosed according to the SISVEFLU database (outpatient clinic or hospital), and 3) likelihood of occurrence of a health outcome (ambulatory discharge, hospitalisation and subsequent discharge, or death).

- Scenario 0: Symptomatic individual who does not seek medical care, and self-medicates with over-the-counter drugs.
- Scenario 1: Symptomatic individual visited an outpatient clinic, had a positive polymerase chain reaction (PCR) result for influenza, was managed only in ambulatory care, and had a complete recovery.
- Scenario 2: Symptomatic individual visited an outpatient clinic, had a positive PCR result for influenza for which, due to the severity, they were referred for hospital care where they had a complete recovery, and were discharged.
- Scenario 3: Symptomatic individual visited an outpatient clinic, had a positive PCR result for influenza and, due to severity, was referred for hospital care, and died.
- Scenario 4: Symptomatic individual visited a hospital emergency room (ER), had a positive PCR result for influenza, was discharged to an outpatient clinic for follow-up, and had a complete recovery.
- Scenario 5: Symptomatic individual visited a hospital ER, had a positive PCR result for influenza, was admitted to hospital for follow-up with non-severe clinical status, and had a complete recovery.
- Scenario 6: Symptomatic individual visited a hospital ER, had a positive PCR result for influenza, was admitted for follow-up with severe clinical status, and had a complete recovery.
- Scenario 7: Symptomatic individual visited a hospital ER, had a positive PCR result for influenza, was admitted for hospital follow-up, and died.

The decision-analytic model used was shown in **Figure 1** of the main content.

For cases from scenario 0 (symptomatic individuals who do not seek medical care), we followed the method of Molinari et al. [1], and considered the likelihood of seeking medical care for patients with low risk (**Table S2.5**).

For estimation of cases for scenarios either from ambulatory or inpatient care that do not end in death (1, 2, 4, 5, and 6), the likelihood of each scenario was calculated using data registered from health monitoring units at SISVEFLU. Within this database, there is a variable named “Evolution” where health professionals from monitoring units register, for each case, the code that corresponds to the health outcome of that case. **Table S2.6** shows the different codes that were found in the database, and the scenario to which they were allocated.

For scenarios either from ambulatory or inpatient care that resulted in death (3 and 7), the number of deaths was obtained directly from SISVEFLU. Hence, following this classification, all 50,900 cases from 9 seasons between 2009 and 2018 were allocated to a single scenario and, based on the date of each case, they were allocated to the appropriate season (from 2009–2010 to 2017–2018; 433 cases that would correspond to season 2018–2019 were not used). Based on that, the likelihood of occurrence of each scenario for a typical influenza season was estimated, and these likelihoods were used to project national estimates of influenza. **Table S2.7** shows classification of cases for each of the SISVEFLU codes previously described, whereas **Table S2.8** shows the estimated number of cases per scenario and season.

Finally, **Table S2.9** shows the likelihood of occurrence of each scenario for a typical season.

**Reference for Text S2**

1. Molinari NA, Ortega-Sanchez IR, Messonnier ML, Thompson WW, Wortley PM, Weintraub E, Bridges CB. The annual impact of seasonal influenza in the US: Measuring disease burden and costs. Vaccine. 2007;25:5086-96.

## Table S2.1. Confirmed cases of influenza per season and institution, all ages

| **INSTITUTION** | **2009–010** | **2010–2011** | **2011–2012** | **2012–2013** | **2013–2014** | **2014–2015** | **2015–2016** | **2016–2017** | **2017–2018** | **TOTAL PER INSTITUTION** |
| --- | --- | --- | --- | --- | --- | --- | --- | --- | --- | --- |
| SSA | 1447 | 2379 | 4677 | 2292 | 4775 | 1838 | 5155 | 3068 | 2173 | 27,804 |
| IMSS | 1008 | 1675 | 1809 | 1000 | 3210 | 1075 | 3470 | 2605 | 1688 | 17,540 |
| ISSSTE | 97 | 45 | 346 | 133 | 818 | 189 | 656 | 550 | 316 | 3150 |
| IMSS-OPORTUNIDADES | 2 | 28 | 48 | 36 | 49 | 33 | 63 | 38 | 27 | 324 |
| PEMEX | 14 | 18 | 3 | 3 | 26 | 8 | 15 | 33 | 33 | 153 |
| SEDENA | 0 | 0 | 2 | 0 | 0 | 0 | 0 | 0 | 0 | 2 |
| SEMAR | 0 | 0 | 0 | 1 | 2 | 0 | 0 | 0 | 0 | 3 |
| DIF | 0 | 0 | 6 | 1 | 13 | 7 | 17 | 6 | 7 | 57 |
| STATE | 2 | 7 | 68 | 20 | 45 | 28 | 129 | 70 | 29 | 398 |
| UNIVERSITY | 18 | 2 | 1 | 4 | 16 | 20 | 45 | 52 | 15 | 173 |
| PRIVATE | 92 | 157 | 79 | 28 | 39 | 44 | 85 | 47 | 54 | 625 |
| NOT IDENTIFIED | 213 | 18 | 5 | 1 | 1 | 0 | 0 | 0 | 0 | 238 |
| **TOTAL PER SEASON** | **2893** | **4329** | **7044** | **3519** | **8994** | **3242** | **9635** | **6469** | **4342** | **50,467** |

Abbreviations: SSA, Secretaría de Salud (Health Secretariat); IMSS, Instituto Mexicano del Seguro Social (Mexican Social Security Institute); ISSSTE, Instituto de Seguridad y Servicios Sociales para los Trabajadores del Estado (Institute for Social Security and Services for State Workers); MoH, Ministry of Health; IMSS – OPORTUNIDADES, Instituto Mexicano del seguro Social – Programa Oportunidades (Mexican Social Security Institute – Oportunities Programme; PEMEX, Petroleos Mexicanos (Mexican State Oil Company), SEDENA, Secretaría de la Defensa Nacional (Secretariat of National Defense); SEMAR, Secretaría de Marina (Secretariat of the Navy); DIF, Desarrollo Integral de la Familia (Programme for the Integral Family Development), STATE, Servicios Estatales de Salud (State Health Services); UNIVERSITY, Servicios Médicos Universitarios (University Health Services); PRIVATE, Servicios de Salud Privados (Private Health Services); NOT IDENTIFIED, No tidentificado (Source not identified).

*Source: SISVEFLU*

## Table S2.2. Incidence of influenza in the United States by age group

| **Age group, years** | **2009–2010** | **2010–2011** | **2011–2012** | **2012–2013** | **2013–2014** | **2014–2015** | **2015–2016** | **2016–2017** | **2017–2018** | **2018–2019*** |
| --- | --- | --- | --- | --- | --- | --- | --- | --- | --- | --- |
| 0–4 | 13.51% | 13.74% | 4.70% | 17.82% | 12.71% | 16.14% | 11.50% | 11.48% | 19.98% | 13.51% |
| 5–17 | 9.60% | 8.22% | 3.71% | 12.42% | 7.42% | 11.90% | 8.09% | 11.08% | 13.99% | 9.60% |
| 18–49 | 7.05% | 5.47% | 2.56% | 8.38% | 9.59% | 6.31% | 7.39% | 6.23% | 10.47% | 7.05% |
| 50–64 | 12.38% | 8.24% | 3.18% | 12.85% | 13.71% | 11.63% | 11.20% | 13.65% | 24.59% | 12.38% |
| ≥65 | 7.17% | 4.52% | 2.33% | 9.71% | 3.82% | 10.12% | 3.46% | 9.01% | 14.37% | 7.17% |
| **Weighted average** | 8.94% | 7.22% | 3.11% | 10.88% | 9.46% | 9.46% | 8.14% | 8.98% | 14.23% | 8.94% |

* This season was projected as an average estimate of seasons 2009–2010 to 2017–2018, and it was used to estimate the economic benefits of influenza immunisation to school-aged population.

*Source: Centers for Disease Control and Prevention. Disease Burden of Influenza. https://www.cdc.gov/flu/about/burden/index.html. Accessed 18 May 2019.*

## Table S2.3. Estimated cases of influenza in Mexico, per season

| **Season** | **Population** | **Estimated cases**  **of influenza** | **Cases**  **per 100,000** |
| --- | --- | --- | --- |
| **2009–2010** | 113,462,004 | 10,121,012 | 8920.18 |
| **2010–2011** | 114,551,762 | 8,289,457 | 7236.43 |
| **2011–2012** | 116,179,714 | 3,617,815 | 3113.98 |
| **2012–2013** | 117,668,241 | 12,792,227 | 10,871.44 |
| **2013–2014** | 119,216,240 | 11,242,231 | 9430.12 |
| **2014–2015** | 120,653,293 | 11,378,804 | 9430.99 |
| **2015–2016** | 122,038,924 | 9,881,013 | 8096.61 |
| **2016–2017** | 123,388,022 | 11,021,963 | 8932.77 |
| **2017–2018** | 124,692,044 | 17,652,432 | 14,156.82 |
| **2018–2019** | 125,262,111 | 11,165,666 | 8913.84 |

## Table S2.4. Estimated cases of influenza in Mexico per season and age group

| **Age group, years** | **2009–2010** | **2010–2011** | **2011–2012** | **2012–2013** | **2013–2014** | **2014–2015** | **2015–2016** | **2016–2017** | **2017–2018** | **2018–2019** |
| --- | --- | --- | --- | --- | --- | --- | --- | --- | --- | --- |
| <1 | 301,939 | 305,261 | 104,246 | 395,243 | 281,770 | 357,460 | 254,564 | 253,924 | 441,711 | 298,294 |
| 1–4 | 1,210,937 | 1,229,880 | 419,053 | 1,584,863 | 1,127,300 | 1,427,447 | 1,016,194 | 1,013,446 | 1,762,785 | 1,190,477 |
| 5–11 | 1,523,948 | 1,300,700 | 586,163 | 1,956,918 | 1,165,989 | 1,865,751 | 1,265,614 | 1,727,674 | 2,173,794 | 1,488,395 |
| 12–19 | 1,594,041 | 1,344,530 | 613,104 | 2,044,572 | 1,425,795 | 1,878,270 | 1,413,979 | 1,758,493 | 2,329,343 | 1,589,118 |
| 20–24 | 708,065 | 555,688 | 263,779 | 872,707 | 1,009,093 | 670,122 | 790,435 | 669,502 | 1,129,345 | 761,670 |
| 25–29 | 657,067 | 513,093 | 242,421 | 799,114 | 922,694 | 613,606 | 726,859 | 619,779 | 1,054,080 | 717,363 |
| 30–34 | 622,072 | 486,370 | 229,588 | 754,672 | 867,403 | 573,752 | 676,092 | 573,684 | 971,974 | 660,469 |
| 35–39 | 575,441 | 453,050 | 215,211 | 711,627 | 822,200 | 545,759 | 643,904 | 545,892 | 922,294 | 623,887 |
| 40–44 | 506,251 | 403,115 | 193,653 | 647,041 | 754,478 | 504,854 | 599,875 | 511,836 | 869,992 | 591,651 |
| 45–49 | 426,232 | 341,004 | 164,906 | 555,644 | 654,332 | 442,654 | 532,041 | 459,140 | 788,652 | 541,341 |
| 50–54 | 620,591 | 426,818 | 170,098 | 708,848 | 780,004 | 682,024 | 677,626 | 851,767 | 1,580,555 | 819,425 |
| 55–59 | 494,139 | 342,152 | 137,293 | 575,685 | 636,591 | 558,511 | 556,028 | 699,749 | 1,299,947 | 675,234 |
| 60–64 | 381,962 | 263,784 | 105,826 | 444,756 | 494,004 | 436,038 | 437,106 | 553,942 | 1,035,580 | 540,622 |
| 65–69 | 170,694 | 111,087 | 59,214 | 254,719 | 103,658 | 284,654 | 101,033 | 273,551 | 453,897 | 235,797 |
| 70–74 | 128,393 | 83,191 | 44,172 | 189,317 | 76,737 | 209,745 | 74,042 | 199,249 | 328,503 | 169,662 |
| 75–79 | 91,810 | 59,419 | 31,450 | 134,218 | 54,149 | 147,323 | 51,785 | 138,828 | 228,072 | 117,335 |
| 80–84 | 57,909 | 37,801 | 20,167 | 86,609 | 35,081 | 95,577 | 33,557 | 89,681 | 146,715 | 75,136 |
| 85–89 | 30,966 | 20,301 | 10,883 | 47,015 | 19,184 | 52,708 | 18,668 | 50,296 | 82,808 | 42,579 |
| 90–94 | 13,027 | 8648 | 4690 | 20,451 | 8402 | 23,202 | 8257 | 22,362 | 37,055 | 19,203 |
| ≥95 | 5526 | 3566 | 1899 | 8208 | 3368 | 9345 | 3353 | 9168 | 15331 | 8,008 |
| **TOTAL** | **10,121,012** | **8,289,457** | **3,617,815** | **12,792,227** | **11,242,231** | **11,378,804** | **9,881,013** | **11,021,963** | **17,652,432** | **11,165,666** |

*Source:* estimation using data from CDC and official population projections

## Table S2.5. Likelihood of seeking medical care by age group (scenario 0)

| **Age group, years** | **Likelihood** |
| --- | --- |
| 0–4 | 0.5309 |
| 5–17 | 0.6814 |
| 18–49 | 0.6827 |
| 50–64 | 0.6664 |
| ≥65 | 0.3262 |

*Fuente:* Molinari NA, Ortega-Sanchez IR, Messonnier ML, Thompson WW, Wortley PM, Weintraub E, Bridges CB. The annual impact of seasonal influenza in the US: measuring disease burden and costs. Vaccine. 2007;25:5086–96

## Table S2.6. Classification of cases in each scenario according to health outcome

| **SISVEFLU Code** | **Description** | **Scenario** |
| --- | --- | --- |
| **Patients who seek ambulatory care** | | |
| DISCHARGE | Patient who sought ambulatory care and was discharged. It is not clear if the patient continued pharmacological treatment after discharge; nonetheless the costs of drug therapy are already executed. | 1 |
| UNDER TREATMENT | Patient who sought ambulatory care, was discharged, and continued treatment. | 1 |
| HOUSEHOLD FOLLOW-UP | Patient who sought ambulatory care, was discharged, and continued treatment with household visits. | 1 |
| FINALISED TREATMENT | Patient who sought ambulatory care, was discharged, and finalised treatment. | 1 |
| REFERRAL | Patient who sought ambulatory care and was referred to a hospital for follow-up | 2 |
| DEATH | Patient who sought ambulatory care and resulted in death. | 3 |
| NA | Patient who sought ambulatory care and whose health outcome is unknown. Given that health monitoring units follow a comprehensive protocol to register the final outcome of each case, it is inferred that these cases did not have any complications, and the outcome was discharge. | 1 |
| **Patients who seek inpatient care** | | |
| UNDER TREATMENT | Patient who sought inpatient care, was discharged, and continued treatment in an ambulatory unit. In this case, we assumed that patients only entered the emergency room and were not hospitalised during their stay. | 1 |
| HOUSEHOLD FOLLOW-UP | Patient who sought inpatient care, was discharged, and was monitored through household visits from primary care or ambulatory units. | 1 |
| DISCHARGE CURATION | Patient who sought inpatient care, began treatment, and was discharged for monitoring through a primary care or ambulatory unit. | 4 |
| DISCHARGE IMPROVEMENT |  |  |
| DISCHARGE TRANSFER |  |  |
| VOLUNTARY DISCHARGE |  |  |
| NON-SEVERE  CASE | Patient who sought inpatient care, was hospitalised, began treatment, and was classified as a non-severe case. Outcome was complete recovery and discharge. | 5 |
| SEVERE CASE | Patient who sought inpatient care, was hospitalised, began treatment, and was classified as a severe case. Outcome was complete recovery and discharge. | 6 |
| REFERRAL | Patient who sought inpatient care, was hospitalised, began treatment, and due to the severity of the case, was referred to a tertiary hospital. Outcome was complete recovery and discharge. | 6 |
| DEATH | Patient who sought inpatient care, was hospitalised, began treatment, and did not respond to it. Outcome was death. | 7 |

*Source:* own elaboration using SISVEFLU codes

## Table S2.7. Health outcomes and classification by scenario, results from SISVEFLU

| **Health outcome** | **Number**  **of cases** | **Total number of cases** | **Scenario** |
| --- | --- | --- | --- |
| **Ambulatory care** | | | |
| DISCHARGE IMPROVEMENT | 1 | 25,229 | 1 |
| UNDER TREATMENT | 16,616 |  |  |
| HOUSEHOLD FOLLOW-UP | 1909 |  |  |
| FINALISED TREATMENT | 6357 |  |  |
| NA | 346 |  |  |
| REFERRAL | 46 | 46 | 2 |
| DEATH | 78 | 78 | 3 |
| **Total ambulatory care** | **25,353** | **25,353** |  |
| **Inpatient care** | | | |
| UNDER TREATMENT | 2 | 3 | 1 |
| HOUSEHOLD FOLLOW-UP | 1 |  |  |
| DISCHARGE CURATION | 302 | 12,293 | 4 |
| DISCHARGE TRANSFER | 123 |  |  |
| VOLUNTARY DISCHARGE | 182 |  |  |
| DISCHARGE IMPROVEMENT | 11,686 |  |  |
| NONSEVERE CASE | 6718 | 6718 | 5 |
| SEVERE CASE | 2795 | 2882 | 6 |
| REFERRAL | 87 |  |  |
| DEATH | 3651 | 3651 | 7 |
| **Total inpatient care** | **25,547** | **25,547** |  |
| **Total number of cases** | **50,900** | |  |

*Source:* own elaboration using SISVEFLU codes

## Table S2.8. Estimated cases of influenza per scenario and season

|  | **Scenario** | | | | | | |
| --- | --- | --- | --- | --- | --- | --- | --- |
| **Season** | **0** | **1** | **2** | **4** | **5** | **6** | **Deaths**  **(3 & 7)** |
| **2009–2010** | 6,474,267 | 1,914,404 | 3241 | 958,763 | 545,703 | 224,635 | 182 |
| **2010–2011** | 5,290,737 | 1,577,671 | 2534 | 781,175 | 456,107 | 181,233 | 55 |
| **2011–2012** | 2,320,830 | 691,955 | 1124 | 334,095 | 192,201 | 77,610 | 328 |
| **2012–2013** | 8,135,812 | 2,419,624 | 4087 | 1,238,101 | 704,024 | 290,579 | 79 |
| **2013–2014** | 7,319,868 | 2,121,004 | 3762 | 1,004,169 | 559,955 | 233,473 | 1091 |
| **2014–2015** | 7,172,402 | 2,136,792 | 3666 | 1,146,200 | 647,845 | 271,898 | 154 |
| **2015–2016** | 6,418,879 | 1,866,334 | 3216 | 886,219 | 499,519 | 206,848 | 865 |
| **2016–2017** | 7,014,713 | 2,052,371 | 3700 | 1,089,272 | 600,812 | 261,095 | 652 |
| **2017–2018** | 11,186,789 | 3,240,140 | 6229 | 1,805,526 | 978,593 | 435,154 | 292 |
| **Average (typical season)** | 7,121,978 | 2,081,201 | 3766 | 1,093,507 | 605,286 | 259,928 | 414 |

## Table S2.9. Likelihood of occurrence of each scenario for a typical season

| **Age group, years** | **0** | **1** | **2** | **4** | **5** | **6** |
| --- | --- | --- | --- | --- | --- | --- |
| <1 | 53.09% | 10.98% | 0.08% | 17.57% | 12.78% | 5.51% |
| 1–4 | 53.09% | 19.66% | 0.01% | 14.75% | 9.56% | 2.93% |
| 5–11 | 68.14% | 20.54% | 0.01% | 5.87% | 4.29% | 1.16% |
| 12–19 | 68.17% | 21.63% | 0.02% | 5.00% | 3.90% | 1.28% |
| 20–24 | 68.27% | 21.50% | 0.04% | 5.73% | 3.45% | 1.01% |
| 25–29 | 68.27% | 20.51% | 0.06% | 6.63% | 3.35% | 1.18% |
| 30–34 | 68.27% | 20.48% | 0.05% | 6.55% | 3.37% | 1.28% |
| 35–39 | 68.27% | 19.54% | 0.03% | 6.83% | 3.77% | 1.55% |
| 40–44 | 68.27% | 18.62% | 0.04% | 7.78% | 3.60% | 1.69% |
| 45–49 | 68.27% | 17.05% | 0.01% | 8.92% | 3.53% | 2.22% |
| 50–54 | 66.64% | 16.76% | 0.05% | 9.81% | 4.40% | 2.33% |
| 55–59 | 66.64% | 14.55% | 0.01% | 10.62% | 5.07% | 3.11% |
| 60–64 | 66.64% | 11.68% | 0.09% | 12.55% | 5.27% | 3.77% |
| 65–69 | 32.62% | 19.04% | 0.13% | 27.99% | 12.71% | 7.51% |
| 70–74 | 32.62% | 14.96% | 0.08% | 32.62% | 12.11% | 7.60% |
| 75–79 | 32.62% | 10.41% | 0.00% | 32.76% | 15.91% | 8.30% |
| 80–84 | 32.62% | 9.54% | 0.00% | 34.33% | 14.78% | 8.73% |
| 85–89 | 32.62% | 8.38% | 0.16% | 32.21% | 16.93% | 9.70% |
| 90–94 | 32.62% | 8.34% | 0.00% | 31.69% | 16.68% | 10.67% |
| ≥95 | 32.62% | 10.37% | 0.00% | 24.88% | 16.59% | 15.55% |

# Supplement 3

## Table S3.1. Unit costs using 2018 prices from the three largest public health care providers in Mexico

| **Item** | **Cost**  **ISSSTE** | **Cost**  **IMSS** | **Cost**  **MoH** |
| --- | --- | --- | --- |
| Outpatient consultation | 370.48 | 733.00 | 98.80 |
| Specialist consultation | 811.53 | 1160.00 | 98.80 |
| Emergency room care | 811.53 | 562.00 | 305.21 |
| PCR | 2380.47 | 1932.43 | 2380.47 |
| Bacteriologic culture | 201.34 | 201.34 | 201.34 |
| Amantadine | 45.00 | 45.00 | 45.00 |
| Oseltamivir | 110.87 | 110.87 | 110.87 |
| Paracetamol | 9.30 | 3.12 | 3.12 |
| Ceftriaxone | 11.87 | 11.89 | 10.67 |
| Hospitalisation bed-day | 2064.12 | 7757.00 | 305.21 |

Abbreviations: IMSS, Instituto Mexicano del Seguro Social (Mexican Social Security Institute); ISSSTE, Instituto de Seguridad y Servicios Sociales para los Trabajadores del Estado (Institute for Social Security and Services for State Workers); MoH, Ministry of Health; PCR, polymerase chain reaction

Data sources for each unit cost are the following:

**ISSSTE**

- Outpatient consultation, specialist consultation, emergency room (ER) care, and hospitalisation bed-day: publicly available data from Mexico’s Ministry of Health (Secretaría de Salud, 2004).
- Amantadine: 2018 drugstore price, sold as an over-the-counter drug; it does not require a prescription and therefore can be purchased directly by the patient.^[[1]](#footnote-2)^
- Oseltamivir: 2018 price through consolidated government purchase.
- Paracetamol and Ceftriaxone: ISSSTE’s 2019 Annual Acquisitions Programme.^[[2]](#footnote-3)^ For paracetamol, the code is 25301688; for ceftriaxone, the code is 25300461.
- PCR and throat swab: 2018 price list InDRE.

**IMSS**

- Outpatient consultation, specialist consultation, ER care, and hospitalisation bed-day: Mexico’s Official Federal Gazette.^[[3]](#footnote-4)^
- Amantadine: 2018 drugstore public price.^2^
- Oseltamivir: 2018 price through consolidated government purchase.
- Paracetamol: IMSS Acquisitions Portal.^[[4]](#footnote-5)^
- Ceftriaxone: IMSS Acquisitions Portal.^[[5]](#footnote-6)^
- PCR and throat swab: 2018 price list InDRE.

**Ministry of Health (MoH)**

- Outpatient consultation, specialist consultation, ER care, and hospitalisation bed-day: publicly available data from Mexico’s Ministry of Health (Secretaría de Salud, 2004).3
- Amantadine: 2018 drugstore public price.^2^
- Oseltamivir: 2018 price through consolidated government purchase.
- Paracetamol: IMSS Acquisitions Portal.^4^
- Ceftriaxone: IMSS Acquisitions Portal.5
- PCR and throat swab: 2018 price list InDRE.

## Table S3.2. Unit cost by public health provider and season

| **Unit cost** | **Season** | | | | | | | | |
| --- | --- | --- | --- | --- | --- | --- | --- | --- | --- |
|  | **2009–2010** | **2010–2011** | **2011–2012** | **2012–2013** | **2013–2014** | **2014–2015** | **2015–2016** | **2016–2017** | **2017–2018** |
| **IMSS** |  |  |  |  |  |  |  |  |  |
| Outpatient consultation | 536.69 | 554.98 | 577.80 | 599.79 | 623.90 | 640.87 | 658.95 | 698.77 | 733.00 |
| Specialist consultation | 849.34 | 878.28 | 914.39 | 949.20 | 987.34 | 1014.20 | 1042.82 | 1105.82 | 1160.00 |
| ER care | 411.49 | 425.51 | 443.01 | 459.87 | 478.35 | 491.36 | 505.23 | 535.75 | 562.00 |
| Hospitalization bed-day | 5679.59 | 5873.12 | 6114.59 | 6347.34 | 6602.41 | 6782.04 | 6973.41 | 7394.71 | 7757.00 |
| Amantadine | 32.95 | 34.07 | 35.47 | 36.82 | 38.30 | 39.34 | 40.45 | 42.90 | 45.00 |
| Oseltamivir | 81.18 | 83.94 | 87.40 | 90.72 | 94.37 | 96.94 | 99.67 | 105.69 | 110.87 |
| Paracetamol | 2.28 | 2.36 | 2.46 | 2.55 | 2.66 | 2.73 | 2.80 | 2.97 | 3.12 |
| Ceftriaxone | 8.71 | 9.00 | 9.37 | 9.73 | 10.12 | 10.40 | 10.69 | 11.33 | 11.89 |
| PCR | 1414.90 | 1463.12 | 1523.27 | 1581.25 | 1644.80 | 1689.55 | 1737.22 | 1842.17 | 1932.43 |
| Bacteriologic culture | 147.42 | 152.44 | 158.71 | 164.75 | 171.37 | 176.03 | 181.00 | 191.94 | 201.34 |
| **ISSSTE** |  |  |  |  |  |  |  |  |  |
| Outpatient consultation | 271.26 | 280.51 | 292.04 | 303.16 | 315.34 | 323.92 | 333.06 | 353.18 | 370.48 |
| Specialist consultation | 594.20 | 614.44 | 639.71 | 664.06 | 690.74 | 709.53 | 729.56 | 773.63 | 811.53 |
| ER care | 594.20 | 614.44 | 639.71 | 664.06 | 690.74 | 709.53 | 729.56 | 773.63 | 811.53 |
| Hospitalization bed-day | 1511.33 | 1562.82 | 1627.08 | 1689.01 | 1756.89 | 1804.68 | 1855.61 | 1967.71 | 2064.12 |
| Amantadine | 32.95 | 34.07 | 35.47 | 36.82 | 38.30 | 39.34 | 40.45 | 42.90 | 45.00 |
| Oseltamivir | 81.18 | 83.94 | 87.40 | 90.72 | 94.37 | 96.94 | 99.67 | 105.69 | 110.87 |
| Paracetamol | 6.81 | 7.04 | 7.33 | 7.61 | 7.92 | 8.13 | 8.36 | 8.87 | 9.30 |
| Ceftriaxone | 8.69 | 8.99 | 9.36 | 9.71 | 10.10 | 10.38 | 10.67 | 11.32 | 11.87 |
| PCR | 1742.95 | 1802.34 | 1876.45 | 1947.87 | 2026.15 | 2081.27 | 2140.00 | 2269.29 | 2380.47 |
| Bacteriologic culture | 147.42 | 152.44 | 158.71 | 164.75 | 171.37 | 176.03 | 181.00 | 191.94 | 201.34 |
| **Ministry of Health (MoH)** | | | | | | | | | |
| Outpatient consultation | 72.34 | 74.80 | 77.88 | 80.84 | 84.09 | 86.38 | 88.82 | 94.18 | 98.80 |
| Specialist consultation | 72.34 | 74.80 | 77.88 | 80.84 | 84.09 | 86.38 | 88.82 | 94.18 | 98.80 |
| ER care | 223.47 | 231.08 | 240.59 | 249.74 | 259.78 | 266.85 | 274.38 | 290.95 | 305.21 |
| Hospitalization bed-day | 223.47 | 231.08 | 240.59 | 249.74 | 259.78 | 266.85 | 274.38 | 290.95 | 305.21 |
| Amantadine | 32.95 | 34.07 | 35.47 | 36.82 | 38.30 | 39.34 | 40.45 | 42.90 | 45.00 |
| Oseltamivir | 81.18 | 83.94 | 87.40 | 90.72 | 94.37 | 96.94 | 99.67 | 105.69 | 110.87 |
| Paracetamol | 2.28 | 2.36 | 2.46 | 2.55 | 2.66 | 2.73 | 2.80 | 2.97 | 3.12 |
| Ceftriaxone | 7.81 | 8.08 | 8.41 | 8.73 | 9.08 | 9.33 | 9.59 | 10.17 | 10.67 |
| PCR | 1742.95 | 1802.34 | 1876.45 | 1947.87 | 2026.15 | 2081.27 | 2140.00 | 2269.29 | 2380.47 |
| Bacteriologic culture | 147.42 | 152.44 | 158.71 | 164.75 | 171.37 | 176.03 | 181.00 | 191.94 | 201.34 |

Data adjusted using Mexico’s National Consumer Price Index (INPC)

Abbreviations: IMSS, Instituto Mexicano del Seguro Social (Mexican Social Security Institute); ISSSTE, Instituto de Seguridad y Servicios Sociales para los Trabajadores del Estado (Institute for Social Security and Services for State Workers); ER, emergency room; PCR, polymerase chain reaction

## Table S3.3. Affiliated population by public health provider and season

| **Season** | **IMSS^a^** | **ISSSTE^b^** | **MoH^c^** |
| --- | --- | --- | --- |
| 2009–2010 | 50,722,198 | 11,791,419 | 37,325,834 |
| 2010–2011 | 53,608,241 | 12,100,042 | 47,671,017 |
| 2011–2012 | 56,191,147 | 12,328,170 | 52,365,663 |
| 2012–2013 | 58,493,930 | 12,540,089 | 54,273,005 |
| 2013–2014 | 59,499,554 | 12,717,193 | 56,469,000 |
| 2014–2015 | 60,676,058 | 12,888,774 | 57,202,811 |
| 2015–2016 | 62,672,649 | 13,043,445 | 56,014,787 |
| 2016–2017 | 64,783,558 | 13,184,870 | 54,214,570 |
| 2017–2018 | 67,122,622 | 13,301,986 | 53,502,114 |

^a^IMSS: IMSS. Memoria Estadística 2018. [2018 Statistical Report] Available via <http://www.imss.gob.mx/conoce-al-imss/memoria-estadistica-2018>

^b^ISSSTE: ISSSTE. Anuario Estadística 2018 [2018 Annual Statistical Report]. Available via <http://www.issste.gob.mx/datosabiertos/anuarios/anuarios2018.html#cap1>

^c^Seguro Popular. Seguro Popular. Beneficiarios de Protección Social en Salud de Seguro Popular. [Popular insurance. Beneficiaries of Social Protection in Popular Health Insurance.] Available via <https://datos.gob.mx/busca/dataset/beneficiarios-de-proteccion-social-en-salud-de-seguro-popular>

Abbreviations: IMSS, Instituto Mexicano del Seguro Social (Mexican Social Security Institute); ISSSTE, Instituto de Seguridad y Servicios Sociales para los Trabajadores del Estado (Institute for Social Security and Services for State Workers); MoH, Ministry of Health

## Table S3.4. Percentage of population by public health provider and per season

(based on affiliated population by public health provider)

| **Season** | **IMSS** | **ISSSTE** | **MoH** |
| --- | --- | --- | --- |
| 2009–2010 | 50.80% | 11.81% | 37.39% |
| 2010–2011 | 47.28% | 10.67% | 42.05% |
| 2011–2012 | 46.48% | 10.20% | 43.32% |
| 2012–2013 | 46.68% | 10.01% | 43.31% |
| 2013–2014 | 46.24% | 9.88% | 43.88% |
| 2014–2015 | 46.40% | 9.86% | 43.74% |
| 2015–2016 | 47.58% | 9.90% | 42.52% |
| 2016–2017 | 49.01% | 9.97% | 41.01% |
| 2017–2018 | 50.12% | 9.93% | 39.95% |

Abbreviations: IMSS, Instituto Mexicano del Seguro Social (Mexican Social Security Institute); ISSSTE, Instituto de Seguridad y Servicios Sociales para los Trabajadores del Estado (Institute for Social Security and Services for State Workers); MoH, Ministry of Health

## Table S3.5. Average unit cost per season (weighted cost per public health provider)

| **Unit cost** | **Season** | | | | | | | | |
| --- | --- | --- | --- | --- | --- | --- | --- | --- | --- |
|  | **2009–2010** | **2010–2011** | **2011–2012** | **2012–2013** | **2013–2014** | **2014–2015** | **2015–2016** | **2016–2017** | **2017–2018** |
| Outpatient consultation | 331.74 | 323.79 | 332.10 | 345.34 | 356.53 | 367.07 | 384.25 | 416.33 | 443.64 |
| Specialist consultation | 528.72 | 512.30 | 524.01 | 544.56 | 561.67 | 578.31 | 606.14 | 657.76 | 701.45 |
| ER care | 362.78 | 363.93 | 375.38 | 389.29 | 403.43 | 414.65 | 429.28 | 459.08 | 484.20 |
| Hospitalization bed-day | 3147.49 | 3040.89 | 3112.41 | 3240.17 | 3340.33 | 3441.46 | 3618.09 | 3939.79 | 4214.67 |
| Amantadine | 32.95 | 34.07 | 35.47 | 36.82 | 38.30 | 39.34 | 40.45 | 42.90 | 45.00 |
| Oseltamivir | 81.18 | 83.94 | 87.40 | 90.72 | 94.37 | 96.94 | 99.67 | 105.69 | 110.87 |
| Paracetamol | 2.82 | 2.86 | 2.96 | 3.06 | 3.18 | 3.26 | 3.35 | 3.56 | 3.73 |
| Ceftriaxone | 8.37 | 8.61 | 8.95 | 9.30 | 9.66 | 9.93 | 10.22 | 10.86 | 11.40 |
| PCR | 1576.29 | 1641.95 | 1712.28 | 1776.73 | 1849.83 | 1899.51 | 1948.37 | 2059.96 | 2155.92 |
| Bacteriologic culture | 147.42 | 152.44 | 158.71 | 164.75 | 171.37 | 176.03 | 181.00 | 191.94 | 201.34 |

Abbreviations: IMSS, Instituto Mexicano del Seguro Social (Mexican Social Security Institute); ISSSTE, Instituto de Seguridad y Servicios Sociales para los Trabajadores del Estado (Institute for Social Security and Services for State Workers); ER, emergency room; PCR, polymerase chain reaction

## Table S3.6. National Consumer Price Index (INPC) at baseline year 2018

| **YEAR** | **INPC** |
| --- | --- |
| 2003 | 54.282578000000 |
| 2004 | 56.827566250000 |
| 2005 | 59.093883916667 |
| 2006 | 61.238675083333 |
| 2007 | 63.667921083333 |
| 2008 | 66.930890416667 |
| 2009 | 70.476458583333 |
| 2010 | 73.405973250000 |
| 2011 | 75.907193333333 |
| 2012 | 79.028124166667 |
| 2013 | 82.036242583333 |
| 2014 | 85.332965250000 |
| 2015 | 87.654569166667 |
| 2016 | 90.127924833333 |
| 2017 | 95.572963583333 |
| 2018 | 100.255418500000 |

2018 constant prices from Mexico’s INCP were used.

*Source:* INEGI (National Institute of Statistics and Geography)

# Supplement 4

## Figure S2. Projection of the school-aged population and total population in Mexico (2010–2030)

School-aged population

Source: Population projections from Consejo Nacional de Población [National Population Council]

## Table S4. Effectiveness of influenza vaccination in the Northern Hemisphere

| **Effectiveness** | **95% CI** | **Source** |
| --- | --- | --- |
| **2009–2010** | | |
| 56% | 23%–75% | Griffin, et al., 2011^1^ |
| **2010–2011** | | |
| 60% | 53%–66% | Treanor et al., 2012^2^ |
| **2011–2012** | | |
| 47% | 36%–56% | Ohmit et al., 2014^3^ |
| 59% | 43%–70% | Skowronski et al., 2014^4^ |
| A(H1N1)pdm09: 42.80% A(H3N2): 38.1% | 6.3%–65.0% | Rondy et al., 2016^5^ |
| **2012–2013** | | |
| 49% | 43%–55% | McLean et al., 2015^6^ |
| A(H3N2): 42.2% A(H1N1)pdm09: 50.4% B: 49.3% | 14.9%–60.7% 28.4%–65.6% 28.4%–65.6% | Kissling et al., 2014^7^ |
| **2013–2014** | | |
| 68% | 58%–76% | Skowronski et al., 2015^8^ |
| 54% | 46%–61% | Gaglani et al., 2016^9^ |
| **2014–2015** | | |
| 19% | 10%–27% | Zimmerman et al., 2016^10^ |
| A(H3N2):14.4% A(H1N1)pdm09: 54.2% B: 48.0% | 6.3%–31% 31.2%–69.6% 28.9%–61.9% | Valenciano et al., 2016^11^ |
| 22% | 8%–33% | Puig-Barberà et al., 2016^12^ |
| **2015–2016** | | |
| 48% | 41%–55% | Jackson et al., 2017^13^ |
| 64% | 44%–77% | Chambers et al., 2016^14^ |
| 32.9% | 15.5%–46.7% | Kissling et al., 2018^15^ |
| **2016–2017** | | |
| 48% | 37%–57% | Flannery et al., 2017^16^ |
| **2017–2018** | | |
| 36% | 27%–44% | Flannery et al. 2017^16^ |
| 42% | 25%–55% | Skowronski et al., 2018^17^ |
| **2018–2019** | | |
| 47% | 34%–57% | Doyle et al., 2019^18^ |

Abbreviation: CI, confidence interval

**References for Table S4:**

1. Griffin MR, Monto AS, Belongia EA, Treanor JJ, Chen Q, Chen J, Talbot HK, Ohmit SE, Coleman LA, Lofthus G, et al. Effectiveness of Non-Adjuvanted Pandemic Influenza A Vaccines for Preventing Pandemic Influenza Acute Respiratory Illness Visits in 4 U. S. Communities. PLoS One. 2011;6(8):e23085. <https://doi.org/10.1371/journal.pone.0023085>
2. Treanor JJ, Talbot HK, Ohmit SE, Coleman LA, Thompson MG, Cheng PY, Petrie JG, Lofthus G, Meece JK, Williams JV, et al. Effectiveness of seasonal influenza vaccines in the United States during a season with circulation of all three vaccine strains. Clin Infect Dis. 2012;55(7):951–959. <https://doi.org/10.1093/cid/cis574>.
3. Ohmit SE, Thompson MG, Petrie JG, Thaker SN, Jackson ML, Belongia EA, Zimmerman RK, Gaglani M, Lamerato L, Spencer SM, et al. Influenza vaccine effectiveness in the 2011-2012 season: Protection against each circulating virus and the effect of prior vaccination on estimates. Clin Infect Dis. 2015;58(3):319–327. <https://doi.org/10.1093/cid/cit736>.
4. Skowronski DM, Janjua NZ, Sabaiduc S, De Serres G, Winter AL, Gubbay JB, Dickinson JA, Fonseca K, Charest H, Bastien N, et al. Influenza A/subtype and B/lineage effectiveness estimates for the 2011-2012 trivalent vaccine: Cross-season and cross-lineage protection with unchanged vaccine. J Infect Dis. 2014;210(1):126–137. <https://doi.org/10.1093/infdis/jiu048>.
5. Rondy M, Castilla J, Launay O, Costanzo S, Ezpeleta C, Galtier F, de Gaetano Donatai K, Moren A. Moderate influenza vaccine effectiveness against hospitalisation with A(H3N2) and A(H1N1) influenza in 2013–14: Results from the InNHOVE network. Hum Vaccin Immunother. 2016;12(5):1217–1224. <https://doi.org/10.1080/21645515.2015.1126013>.
6. McLean HQ, Thompson MG, Sundaram ME, Kieke BA, Gaglani M, Murthy K, Piedra PA, Zimmerman RK, Nowalk MP, Raviotta JM, et al. Influenza vaccine effectiveness in the United States during 2012-2013: Variable protection by age and virus type. J Infect Dis. 2015;211(10):1529–1540. <https://doi.org/10.1093/infdis/jiu647>.
7. Kissling E, Valenciano M, Buchholz U, Larrauri A, Cohen JM, Nunes B, Rogalska J, Pitigoi D, Paradowska-Staniewicz I, Reuss A, et al. Influenza vaccine effectiveness estimates in Europe in a season with three influenza type/subtypes circulating: the I-MOVE multicentre case-control study, influenza season 2012/13. Euro Surveill. 2014;19(6):pii=20701. https://doi.org/10.2807/1560-7917.ES2014.19.6.20701
8. Skowronski DM, Chambers C, Sabaiduc S, De Serres G, Winter AL, Dickinson JA, Gubbay J, Fonseca K, Charest H, Krajden M, et al. Integrated sentinel surveillance linking genetic, antigenic, andepidemiologic monitoring of influenza vaccine-virus relatedness and effectiveness during the 2013-2014 influenza season. J Infect Dis. 2015;212(5):726–739. <https://doi.org/10.1093/infdis/jiv177>.
9. Gaglani M, Pruszynski J, Murthy K, Clipper L, Robertson A, Reis M, Chung JR, Piedra PA, Avadhanula V, Nowalk MP, et al. Influenza Vaccine Effectiveness Against 2009 Pandemic Influenza A(H1N1) Virus Differed by Vaccine Type during 2013-2014 in the United States. Journal of Infectious Diseases. J Infect Dis. 2016;213(10):1546–1556. <https://doi.org/10.1093/infdis/jiv577>
10. Zimmerman RK, Nowalk MP, Chung J, Jackson ML, Jackson LA, Pietre JG, Monto AS, McLean HQ, Belongia EA, Gaglani M, et al. 2014-2015 Influenza Vaccine Effectiveness in the United States by Vaccine Type. Clin Infect Dis. 2016;63(12):1564–1573. <https://doi.org/10.1093/cid/ciw635>.
11. Valenciano M, Kissling E, Reuss A, Rizzo C, Gherasim A, Horvath JK, Domegan L, Pitigoi D, Machado A, Pradowska-Stankiewicz IA, et al. Vaccine effectiveness in preventing laboratory-confirmed influenza in primary care patients in a season of co-circulation of influenza A(H1N1)pdm09, B and drifted A(H3N2), I-MOVE multicentre case–control study, Europe 2014/15. Euro Surveill. 2016;21(7):pii=30139. <https://doi.org/10.2807/1560-7917.ES.2016.21.7.30139>.
12. Puig-Barberà J, Burtseva E, Yu H, Cowling BJ, Badur S, Kyncl J, Sominina A, GIHSN. Influenza epidemiology and influenza vaccine effectiveness during the 2014-2015 season: annual report from the Global Influenza Hospital Surveillance Network. BMC Public Health. 2016;16(Suppl 1):757. <https://doi.org/10.1186/s12889-016-3378-1>
13. Jackson ML, Chung JR, Jackson LA, Phillips CH, Benoit J, Monto AS, Martin ET, Belongia EA, McLean HQ, Galgani M, et al. Influenza Vaccine Effectiveness in the United States during the 2015-2016 Season. N Engl J Med. 2017;377(6):534–543. <https://doi.org/10.1056/NEJMoa1700153>
14. Chambers C, Skowronski DM, Sabaiduc S, Winter AL, Dickinson JA, De Serres G, Gubbay JB, Drews SJ, Martineau C, Eshaghi A, et al. Interim estimates of 2015/16 vaccine effectiveness against influenza A(H1N1)pdm09, Canada, February 2016. Euro Surveill. 2016 ;21(11) :30168. <https://doi.org/10.2807/1560-7917.ES.2016.21.11.30168>.
15. Kissling E, Valenciano M, Pozo F, Vilcu AM, Reuss A, Rizzo C, Larrauri A, Horvath JK, Brytting M, Domegan L, et al. 2015/16 I‐MOVE/I‐MOVE+ multicentre case‐control study in Europe: Moderate vaccine effectiveness estimates against influenza A(H1N1)pdm09 and low estimates against lineage‐mismatched influenza B among children. Influenza Other Respi Viruses. 2018;12:423–437. <https://doi.org/10.1111/irv.12520>.
16. Flannery B, Chung JR, Thaker SN, Monto AS, Martin ET, Belongia EA, McLean HQ, Gaglani, M, Murthy K, Zimmerman RK, et al. Interim Estimates of 2016–17 Seasonal Influenza Vaccine Effectiveness — United States, February 2017. MMWR Morb Mortal Wkly Rep. 2017;66(6):167–171.
17. Skowronski DM, Chambers C, De Serres G, Dickinson JA, Winter AL, Hickman R, Chan T, Jassem AN, Drews SJ, Charest H, et al. Early season co-circulation of influenza A(H3N2) and B(Yamagata): interim estimates of 2017/18 vaccine effectiveness, Canada, January 2018. Euro Surveill. 2018;23(5):1–7. <https://doi.org/10.2807/1560-7917.ES.2018.23.5.18>.
18. Doyle JD, Chung JR, Kim SS, Gaglani M, Raiyani C, Zimmerman RK, Nowalk MP, Jackson ML, Jackson LA, Monto AS, et al. Interim Estimates of 2018–19 Seasonal Influenza Vaccine Effectiveness — United States, February 2019. MMWR Morb Mortal Wkly Rep. 2019;68(6):135–139. <https://doi.org/10.15585/mmwr.mm6806a2>.

# Supplement 5

## Table S5.1. Confirmed influenza cases by age group and season

| **Age group, years** | **2009–2010** | **2010–2011** | **2011–2012** | **2012–2013** | **2013–2014** | **2014–2015** | **2015–2016** | **2016–2017** | **2017–2018** | **2018–2019** | **TOTAL** | **%** |
| --- | --- | --- | --- | --- | --- | --- | --- | --- | --- | --- | --- | --- |
| **0–4** | 382 | 663 | 758 | 543 | 949 | 441 | 1150 | 899 | 695 | 37 | **6517** | **12.80** |
| **5–11** | 259 | 589 | 756 | 454 | 655 | 309 | 931 | 660 | 466 | 42 | **5121** | **10.06** |
| **12–17** | 205 | 317 | 562 | 279 | 461 | 223 | 507 | 315 | 221 | 9 | **3099** | **6.09** |
| **18–49** | 1562 | 2057 | 3733 | 1558 | 4605 | 1360 | 4468 | 2621 | 1823 | 89 | **23,876** | **46.91** |
| **50–59** | 284 | 305 | 722 | 307 | 1228 | 334 | 1209 | 839 | 463 | 34 | **5725** | **11.25** |
| **≥60** | 201 | 398 | 513 | 378 | 1096 | 575 | 1370 | 1135 | 857 | 39 | **6562** | **12.89** |
| **TOTAL** | **2893** | **4329** | **7044** | **3519** | **8994** | **3242** | **9635** | **6469** | **4525** | **250** | **50,900** | **100.00** |

*Source:* SISVEFLU

## Table S5.2. Estimated influenza cases by age group and season

| **Age group, years** | **2010–2011** | **2011–2012** | **2012–2013** | **2013–2014** | **2014–2015** | **2015–2016** | **2016–2017** | **2017–2018** | **2018–2019** | **TOTAL** |
| --- | --- | --- | --- | --- | --- | --- | --- | --- | --- | --- |
| **0–4** | 1,541,921 | 526,212 | 1,991,434 | 1,417,562 | 1,794,872 | 1,275,233 | 1,268,274 | 2,197,168 | 1,476,442 | **13,489,116** |
| **5–11** | 1,291,012 | 583,585 | 1,952,068 | 1,164,843 | 1,865,513 | 1,266,616 | 1,729,655 | 2,176,925 | 1,491,125 | **13,521,341** |
| **12–17** | 1,100,268 | 496,595 | 1,658,823 | 989,697 | 1,586,659 | 1,080,209 | 1,480,577 | 1,869,632 | 1,283,742 | **11,546,202** |
| **18–49** | 2,982,160 | 1,421,706 | 4,716,432 | 5,471,988 | 3,646,289 | 4,320,570 | 3,679,992 | 6,247,656 | 4,245,921 | **36,732,715** |
| **50–59** | 777,680 | 312,180 | 1,308,217 | 1,445,898 | 1,267,128 | 1,259,132 | 1,581,055 | 2,928,240 | 1,515,030 | **12,394,559** |
| **≥60** | 582,078 | 275,863 | 1,176,698 | 791,211 | 1,253,279 | 727,139 | 1,335,003 | 2,329,457 | 1,211,712 | **9,682,439** |
| **TOTAL** | **8,275,119** | **3,616,140** | **12,803,672** | **11,281,198** | **11,413,739** | **9,928,898** | **11,074,556** | **17,749,078** | **11,223,972** | **97,366,372** |
| **Population** | **114,551,762** | **116,179,714** | **117,688,241** | **119,216,240** | **120,653,293** | **122,038,924** | **123,388,002** | **124,692,044** | **125,960,168** |  |
| **Attack rate (%)** | **7.22** | **3.11** | **10.88** | **9.46** | **9.46** | **8.14** | **8.98** | **14.23** | **8.91** |  |

## Table S5.3. Incidence per 100,000 inhabitants by age group and season

| **Age group, years** | **2009–2010** | **2010–2011** | **2011–2012** | **2012–2013** | **2013–2014** | **2014–2015** | **2015–2016** | **2016–2017** | **2017–2018** | **2018–2019** | **Average** |
| --- | --- | --- | --- | --- | --- | --- | --- | --- | --- | --- | --- |
| **0–4** | 3.40 | 5.91 | 6.77 | 4.86 | 8.51 | 3.96 | 10.37 | 8.14 | 6.32 | 0.34 | **5.86** |
| **5–11** | 1.65 | 3.75 | 4.81 | 2.89 | 4.17 | 1.97 | 5.95 | 4.23 | 2.99 | 0.27 | **3.27** |
| **12–17** | 1.53 | 2.37 | 4.20 | 2.09 | 3.45 | 1.67 | 3.80 | 2.36 | 1.65 | 0.07 | **2.32** |
| **18–49** | 2.91 | 3.77 | 6.73 | 2.77 | 8.07 | 2.35 | 7.64 | 4.44 | 3.05 | 0.15 | **4.19** |
| **50–59** | 3.13 | 3.23 | 7.36 | 3.02 | 11.65 | 3.06 | 10.75 | 7.25 | 3.89 | 0.28 | **5.36** |
| **≥60** | 2.03 | 3.88 | 4.83 | 3.44 | 9.61 | 4.86 | 11.18 | 8.93 | 6.50 | 0.29 | **5.55** |
| **TOTAL** | **2.56** | **3.78** | **6.06** | **2.99** | **7.54** | **2.69** | **7.90** | **5.24** | **3.63** | **0.20** | **4.26** |

*Source:* SISVEFLU

## Table S5.4. Lethality by age group and season

| **Age group, years** | **2009–2010** | **2010–2011** | **2011–2012** | **2012–2013** | **2013–2014** | **2014–2015** | **2015–2016** | **2016–2017** | **2017–2018** | **2018–2019** | **Average** |
| --- | --- | --- | --- | --- | --- | --- | --- | --- | --- | --- | --- |
| **0–4** | 1.83% | 0.45% | 3.43% | 1.47% | 5.48% | 1.36% | 4.61% | 3.45% | 2.16% | 8.11% | **3.13%** |
| **5–11** | 1.54% | 0.00% | 0.93% | 0.44% | 2.60% | 0.97% | 2.58% | 1.52% | 1.72% | 0.00% | **1.46%** |
| **12–17** | 1.95% | 0.32% | 1.25% | 0.36% | 2.39% | 1.79% | 1.58% | 0.95% | 2.26% | 0.00% | **1.42%** |
| **18–49** | 7.23% | 0.92% | 3.16% | 1.93% | 10.49% | 2.94% | 6.85% | 8.36% | 4.61% | 7.87% | **5.94%** |
| **50–59** | 12.32% | 2.30% | 10.53% | 3.58% | 21.82% | 7.19% | 17.62% | 16.21% | 12.31% | 17.65% | **14.55%** |
| **≥60** | 9.45% | 6.28% | 18.32% | 7.14% | 23.72% | 13.39% | 19.05% | 22.29% | 14.47% | 5.13% | **17.40%** |
| **TOTAL** | 6.29% | 1.27% | 4.66% | 2.24% | 12.13% | 4.75% | 8.98% | 10.08% | 6.48% | 7.20% | **7.30%** |

*Source:* SISVEFLU

## Table S5.5. Years of life lost per age group by season

| **Age group, years** | **2009–2010** | **2010–2011** | **2011–2012** | **2012–2013** | **2013–2014** | **2014–2015** | **2015–2016** | **2016–2017** | **2017–2018** | **2018–2019** | **Total** | **%** |
| --- | --- | --- | --- | --- | --- | --- | --- | --- | --- | --- | --- | --- |
| **0–4** | 366 | 151 | 1847 | 604 | 2831 | 458 | 3053 | 1408 | 1053 | 154 | **11,925** | 12.47% |
| **5–11** | 284 | 0 | 460 | 132 | 1149 | 207 | 1656 | 664 | 549 | 0 | **5101** | 5.33% |
| **12–17** | 236 | 62 | 431 | 61 | 685 | 240 | 494 | 186 | 314 | 0 | **2709** | 2.83% |
| **18–49** | 4425 | 709 | 4479 | 1140 | 17,292 | 1596 | 10,715 | 7747 | 3128 | 247 | **51,478** | 53.84% |
| **50–59** | 747 | 131 | 1498 | 215 | 5388 | 529 | 4319 | 2785 | 1198 | 131 | **16,941** | 17.72% |
| **≥60** | 154 | 115 | 716 | 144 | 1960 | 372 | 1697 | 1682 | 617 | 9 | **7466** | 7.81% |
| **TOTAL** | **6212** | **1168** | **9431** | **2296** | **29,305** | **3402** | **21,934** | **14,472** | **6859** | **541** | **95,620** | 100.00% |

*Source:* SISVEFLU

## Table S5.6. Estimated influenza cases by age and season

| **Age, years** | **2010–2011** | **2011–2012** | **2012–2013** | **2013–2014** | **2014–2015** | **2015–2016** | **2016–2017** | **2017–2018** | **2018–2019** | **Total** |
| --- | --- | --- | --- | --- | --- | --- | --- | --- | --- | --- |
| **5** | 185,270 | 83,455 | 278,491 | 165,983 | 265,372 | 180,070 | 246,032 | 309,937 | 212,304 | **1,926,913** |
| **6** | 185,465 | 83,641 | 278,896 | 166,114 | 265,851 | 180,321 | 246,243 | 310,059 | 212,542 | **1,929,133** |
| **7** | 184,967 | 83,733 | 279,550 | 166,366 | 266,094 | 180,666 | 246,609 | 310,352 | 212,642 | **1,930,978** |
| **8** | 184,380 | 83,511 | 279,882 | 166,769 | 266,527 | 180,854 | 247,102 | 310,838 | 212,859 | **1,932,723** |
| **9** | 184,019 | 83,250 | 279,165 | 166,983 | 267,201 | 181,169 | 247,378 | 311,483 | 213,207 | **1,933,854** |
| **10** | 183,614 | 83,089 | 278,309 | 166,568 | 267,565 | 181,642 | 247,822 | 311,847 | 213,660 | **1,934,115** |
| **11** | 183,297 | 82,905 | 277,775 | 166,060 | 266,904 | 181,894 | 248,470 | 312,408 | 213,911 | **1,933,625** |
| **Total** | **1,291,012** | **583,585** | **1,952,068** | **1,164,843** | **1,865,513** | **1,266,616** | **1,729,655** | **2,176,925** | **1,491,125** | **13,521,341** |

*Source:* SISVEFLU

1. <https://www.farmaciasanpablo.com.mx/medicamentos/genericos/a---b---c---d/clorhidrato-de-amantadina-50-mg-clorfenamina-3-mg-paracetamol-300-mg/p/000000000060160010>) [↑](#footnote-ref-2)
2. <http://www.issste.gob.mx/images/downloads/instituto/administracion/paaas_2019.pdf> [↑](#footnote-ref-3)
3. ACUERDO ACDO.AS3.HCT.291117/275.P.DF y Anexos, dictado por el H. Consejo Técnico en la sesión ordinaria celebrada el día 29 de noviembre de dos mil diecisiete, relativo a la Aprobación de los Costos Unitarios por Nivel de Atención Médica actualizados al año 2018 [dictated by H. Technical Council in ordinary session held on 29 November 2017 on the Approval of Unit Costs by Level of Medical Care, updated 2018] (available via <http://www.dof.gob.mx/nota_detalle.php?codigo=5509604&fecha=28/12/2017> [↑](#footnote-ref-4)
4. <http://compras.imss.gob.mx/?P=imsscomprofich&f=22409921&pr=> [↑](#footnote-ref-5)
5. <http://compras.imss.gob.mx/?P=imsscomprofich&f=22409744&pr=> [↑](#footnote-ref-6)
